# Chromatin structural gene expression stratifies cardiac cell populations in health and disease

**DOI:** 10.1080/15592294.2025.2566505

**Published:** 2025-10-21

**Authors:** Xiaoxiao Geng, Rujula Pradeep, Riley Porter, Lucia García-Gutiérrez, Min Xie, Adam R. Wende, Jianyi Zhang, Isidoro Cobo, Thanh Nguyen, Manuel Rosa-Garrido

**Affiliations:** a Department of Biomedical Engineering, Heersink School of Medicine and School of Engineering, University of Alabama at Birmingham, Birmingham, AL, USA; b Division of Clinical Immunology & Rheumatology, Department of Medicine, Heersink School of Medicine, University of Alabama at Birmingham, Birmingham, AL, USA; # Comprehensive Arthritis, Musculoskeletal, Bone and Autoimmunity Center, University of Alabama at Birmingham, Birmingham, AL, USA; d Department of Molecular Biology, University of Cantabria, Santander, Spain; e Division of Cardiovascular Disease, Department of Medicine, University of Alabama at Birmingham, Birmingham, AL, USA; f Division of Molecular Cellular Pathology, Department of Pathology, University of Alabama at Birmingham, Birmingham, AL, USA; g Department of Cellular and Molecular Signalling, The Institute of Biomedicine and Biotechnology of Cantabria, CSIC-University of Cantabria, Santander, Spain

**Keywords:** Chromatin structure, HMGN3, cardiac disease, snRNA-seq, ChIP-seq

## Abstract

Chromatin structure plays a central role in regulating gene expression and maintaining cellular identity, yet the structural factors driving these processes in cardiac disease remain poorly defined. To investigate whether these factors can distinguish healthy from diseased cardiac cell populations, we generated a comprehensive list of chromatin structural genes based on an extensive literature review. Applying this list to a published single-nuclei RNA sequencing dataset from human hearts with and without dilated cardiomyopathy (DCM), we found that chromatin structural gene expression effectively stratified cardiomyocyte and fibroblast populations by disease status. Diseased cardiomyocytes exhibited reduced expression of contractile genes and increased expression of cardiomyopathy markers, while fibroblasts showed enhanced activation signatures. Among these factors, HMGN3 emerged as a candidate of interest, showing consistent downregulation in cardiomyocytes from DCM human patients, as well as in mouse (pressure overload) and pig (myocardial infarction) models of heart failure. Functional studies in AC16 cells revealed that HMGN3 depletion promoted apoptosis, induced significant changes in gene expression, and reorganized chromatin structure by altering the distribution of the H3K27ac histone mark. These findings identify HMGN3 as a potential regulator of chromatin architecture in diseased cardiomyocytes, highlight the utility of chromatin structural changes in distinguishing pathological cardiac states, and reinforce the role of chromatin organization in shaping the cardiac phenotype.

## Introduction

The structure of chromatin is dynamic, constantly undergoing remodelling to meet the complex needs of the cell. Chromatin topol ogy can be categorized into two levels of organization: global and local. The first, known as higher-order chromatin structure [1], refers to the organization of chromatin beyond the nucleosome level, including the arrangement of chromatin fibres into more complex structures such as chromatin loops, topologically associated domains (TADs), and A/B compartments. The second, referred to as chromatin accessibility [2], describes how open or closed chromatin is to regulatory proteins like transcription factors, RNA polymerase, and other chromatin-binding factors. Both concepts are interconnected, as changes in higher-order chromatin structure can influence chromatin accessibility. However, while higher-order chromatin structure is largely regulated by global chromatin remodelling complexes and the activity of CTCF and cohesins [3], chromatin accessibility is primarily controlled by local factors such as post-translational histone modifications, nucleosome positioning, and chromatin-binding proteins [4]. Together, these two levels of chromatin organization establish a highly regulated framework that dictates gene expression patterns by determining when and where genes are accessible for transcription. Higher-order chromatin structures, such as TADs and chromatin loops, bring enhancers, promoters, and other regulatory elements into proximity, enabling precise control of gene activation and repression. Meanwhile, chromatin accessibility fine-tunes this regulation by determining whether transcriptional machinery can physically interact with DNA. For instance, even if an enhancer is spatially close to its target gene due to chromatin looping, transcription will not occur unless the local chromatin environment is accessible. Disruptions in either of these regulatory layers can lead to aberrant gene expression, contributing to developmental pathology [5].

Our previous studies have linked cardiac disease-one of the leading causes of mortality worldwide – to significant alterations in both global and local chromatin organization [6,7]. These findings suggest that chromatin structure, along with the expression of key chromatin-topology regulators such as CTCF [6,8] and HMGB2 [9], plays a crucial role in shaping the transcriptional landscape and therefore the phenotypic fate of cardiac cells. Single-cell and Single-nuclei RNA sequencing (scRNA-seq, snRNA-seq) experiments using heart tissue have provided new insights into how transcriptional landscapes shift in response to disease progression. In cardiomyocytes, scRNA-seq and snRNA-seq have revealed transcriptional heterogeneity and disease-specific subpopulations marked by metabolic shifts, sarcomeric gene dysregulation, and activation of stress-response pathways. These changes are often accompanied by alterations in chromatin accessibility, suggesting a direct link between chromatin structure and the cell identity diversification under pathological conditions [10,11]. Beyond cardiomyocytes, epigenetic regulation also influences fibroblast and endothelial cell behaviour. scRNA-seq studies have identified fibroblast subsets that exhibit distinct chromatin landscapes, driving maladaptive remodelling and fibrosis. Similarly, endothelial cell populations show transcriptional shifts that correlate with chromatin accessibility changes, affecting angiogenesis and vascular integrity [12–14].

Among the key regulators of chromatin accessibility is the High Mobility Group Nucleosome-binding (HMGN) protein family, a group of non-histone chromatin architectural proteins that regulate chromatin structure by modulating nucleosome dynamics [15]. HMGN proteins antagonize the binding of linker histone H1 in the nucleosome, reducing chromatin compaction and promoting a more accessible chromatin state [16]. By doing so, HMGNs play a crucial role in maintaining the delicate balance between chromatin structure and accessibility, thereby influencing gene expression. The HMGN family consists of five members – HMGN1, HMGN2, HMGN3, HMGN4, and HMGN5—which are ubiquitously expressed in vertebrates, with HMGN1 and HMGN2 being the most abundant. These proteins do not bind specific DNA sequences but preferentially localize to chromatin regulatory elements, where they exert transcriptional regulatory functions [17]. Given their role in chromatin accessibility, HMGN proteins are critical players in various biological processes. HMGN1 and HMGN2 are implicated in embryogenesis [18], neuronal and eye development [19], as well as the differentiation of myoblasts, osteoblasts, and monocytes [20,21]. HMGN3 is involved in early embryonic development [22], ocular development [23], and astrocyte differentiation [24]. HMGN5 plays a role in placenta and heart development [25,26] and influences liver function [27]. At the pathological level, while mouse models with altered HMGN levels survive, HMGNs have been shown to regulate the progression of various diseases. HMGN1 and HMGN2 exhibit both oncogenic and tumour-suppressive properties depending on the cancer type. HMGN1 has also been linked to Down syndrome [28], renal fibrosis [29], and pulmonary hypertension [30], while HMGN2 has demonstrated antimicrobial activity [31] and a protective role against microcephaly [32]. HMGN3 deficiency promotes a diabetic phenotype, suggesting a protective role against this disease [33,34]. HMGN4 serves as a cancer marker for hepatocellular carcinoma [35] and has oncogenic activity in thyroid tumours [36]. HMGN5 has demonstrated tumorigenic activity in bladder [37], pancreatic [38], breast [39], and prostate cancers [40], as well as osteosarcoma [41] and carcinoma [42]. Additionally, HMGN5 overexpression leads to heterochromatin loss, which causes nuclear deformation and disruption of laminin in cardiomyocytes, increasing their size and promoting cardiac hypertrophy and dysfunction [26].

Given the growing evidence implicating chromatin remodelling in the regulation of gene expression, we investigated whether cardiac snRNA-seq data can be effectively clustered based on the differential expression of a selected set of chromatin structural genes. Furthermore, this manuscript explores the potential role of HMGN3 in cardiac physiology, with a particular focus on its involvement in chromatin organization. By examining the molecular mechanisms through which HMGN3 modulates chromatin structure and gene expression, we aim to provide new insights into its impact on cardiac function.

## Materials and methods

### Single RNA-seq bioinformatics analysis

#### Single-nuclei RNA sequencing (snRNA-seq) datasets

Human heart snRNA-seq data (GSE183852) [11] was obtained from the Gene Expression Omnibus, comprising 220,072 nuclei from 25 healthy donors and 13 dilated cardiomyopathy patients. Additionally, a mouse dataset (GSE120064) [43] with 11,492 nuclei, collected from mice subjected to either SHAM surgery or transverse aortic constriction (TAC) injury after an 11-week follow-up, was used to confirm changes in the expression of target genes.

#### Single-nuclei RNA-seq processing and cell-type identification

Following a thorough evaluation of multiple datasets, we validated the high quality of the selected snRNA-seq data for our analyses (Suppl. Figs. S1, 2). Although the dataset included a balanced number of male (54%) and female (46%) donors (Suppl. Fig. S3), nuclei were clustered based on transcriptomic similarity rather than donor sex. As a result, we cannot control for the sex of individual nuclei, and some clusters may be biased towards one sex. For this reason, we routinely exclude sex-biased genes (e.g., XIST, UTY, KDM5D) from our analyses. snRNA-seq data integration, normalization, quality control, doublet filtering, and cell type identification were performed using a general Autoencoder, as previously described [44–46]. Briefly, genes expressed in fewer than 1% of the cells were temporarily filtered out. The remaining gene expression data were processed through a three-layer Autoencoder. The first layer (input) contained the original expression data, the second (embedded) compressed the data into 10 dimensions, and the third (output) reconstructed the embedded data. Once the Autoencoder was optimized to maximize similarity between the input and output layers, the high-dimensional snRNA-seq data could be effectively reduced to just 10 dimensions. These low-dimensional representations were subsequently analysed using Uniform Manifold Approximation and Projection (UMAP) [47,48] and the Density-Based Spatial Clustering of Applications with Noise (DBSCAN) [47–50] algorithms. Cell-type specificity of clusters was determined based on the expression of lineage-specific markers: cardiomyocytes (*ACTC1, MYH7, TNNT2, RYR2*); cardiac endothelial cells (*PECAM1*); cardiac smooth muscle cells (*GJC1, ACTA2*); macrophages (*CD163*); lymphocytes (*CD3E, CD3G, CD8A*); and cardiac fibroblasts (*COL1A1, FN1*).

#### Chromatin-structure-specific analysis of each cell type

After isolating the snRNA-seq data from the entire dataset, the expression levels of our generated list of chromatin structure regulators (Supplementary Table 1) were input into a chromatin-structure-specific Autoencoder. Using the same computational method as described in our previous work [45,51], the Autoencoder embedded the gene expression data into 10 dimensions. The embedded data were then visualized and clustered using the UMAP toolkit [47,48], revealing subclusters within each cell type. Upregulated genes for each cluster were identified using the following criteria: 1) cluster p-value < 10^−6^ (Wilcoxon rank-sum test) [52]. 2) expression in at least 20% of cells within the cluster, and 3) mean abundance at least 2-fold greater in the cluster compared to other clusters. The list of upregulated genes was analysed using the DAVID functional annotation tool [53] to identify enriched pathways and biological processes. Only terms from manually curated Gene Ontology [54], KEGG [55], and Reactome [56] categories were selected. To reduce false discoveries due to multiple hypothesis testing, results were filtered for *p* < 0.01 or Benjamini-adjusted [57] *p* < 0.05. The enrichment score for each pathway and biological process was calculated as the base-10 logarithm of the Benjamini-adjusted p-value.

### Animals and tissue source

C57BL/6J mice were purchased from Jackson Laboratories. Adult Sinclair pigs were obtained through the UAB Animal Resources Program from Prestage Farms MS, Inc. All animals were housed in positive-pressure rooms; mice were kept in microisolator cages, and both species were provided with sterile food, water, and bedding, in accordance with UAB institutional animal care and the American Veterinary Medical Association (AVMA) use guidelines under the UAB Animal Project Number (APN): IACUC-22570. Human heart tissue samples were obtained from the UAB Comprehensive Cardiovascular Center Biobank at the Heersink School of Medicine. All specimens were de-identified to ensure patient privacy and maintain ethical research standards in accordance with the principles outlined in the Declaration of Helsinki. The study was conducted with approval from the UAB Office of the Institutional Review Board (protocol number IRB-300010478).

### Transverse aortic constriction

Transverse aortic constriction (TAC) was performed as previously described [6,58]. Briefly, 10-week-old C57BL/6J mice were anesthetized using isoflurane and the fur was removed with depilatory cream, and placed in the supine position on a heating pad (37°C). A horizontal skin incision (~1 cm) was made at the level of the suprasternal notch, followed by an ~ 3-mm longitudinal cut in the proximal sternum. TAC was induced by placing a metal clip, calibrated to a 30-gauge-diameter needle, between the innominate artery and the left common carotid artery. The muscle, sternum, and skin are closed by suture, the mice are given an injection of buprenorphine (0.05 mg/kg) and allowed to recover on a warming pad until they are fully awake. The SHAM procedure was identical except that the aortic arch was not constricted. Following surgery, mice were monitored weekly by echocardiography and considered to be in heart failure when the ejection fraction (EF) was significantly reduced compared to the mean of the SHAM group. After diagnosis of heart failure (11 weeks post-surgery), the mice were sacrificed by administration of a lethal dose of sodium pentobarbital, followed by potassium chloride, in accordance with AVMA guidelines and with approval from the Institutional Animal Care and Use Committee of the University of Alabama at Birmingham. Cervical dislocation is performed post-euthanasia to confirm complete culling.

### Myocardial infarction

Acute myocardial infarction was induced on postnatal day 14 swine by permanent ligation of the left anterior descending (LAD) coronary artery, as previously described [59]. Briefly, pigs were anesthetized with isoflurane and placed in dorsal recumbency on a heating pad. A midline sternotomy was performed to access the heart, and the LAD artery was ligated distal to the second diagonal branch using a surgical suture. The sternum was then reapproximated, the thoracic cavity was closed in anatomical layers, and residual air was evacuated from the mediastinum. Following the procedure, animals were housed in a temperature-controlled incubator until they could independently regulate their body temperature. Cardiac function was assessed immediately before surgery and at 7 and 28 days afterwards via transthoracic echocardiography. After the diagnosis of the condition, pigs are euthanized using a lethal dose of sodium pentobarbital administered intravenously or intraperitoneally, in accordance with AVMA guidelines and with approval from the Institutional Animal Care and Use Committee.

### Cardiomyocyte isolation

Cardiac tissue from human donors and swine hearts was meticulously sectioned into 1 mm cubes. The tissue was then enzymatically digested for 30 minutes with constant rotation at 37°C using a 50/50 mixture of collagenase B and D (MilliporeSigma, #11088807001, #11088858001) to effectively dissociate the cardiomyocytes from the surrounding connective tissue. The resulting supernatant was filtered and centrifuged at 1000 g for 5 min to enrich for cardiomyocytes. Mouse cardiomyocytes were isolated using a Langendorff apparatus as previously described [6,7]. Briefly, adult mice were heparinized (100 USP units) and anesthetized with sodium pentobarbital (100 µL of 50 mg/mL dilution, administered intraperitoneally). Hearts were excised, mounted on the Langendorff system, and perfused with Tyrode’s solution for 5 minutes at 37°C. The hearts were then perfused with 30 mL of Tyrode’s solution containing collagenase type II and protease type XIV for 15–30 minutes, followed by a wash with oxygenated Krebs buffer (95% O2, 5% CO2) for 10 minutes. Cardiomyocytes were dissociated in Krebs buffer, filtered through a 100-µm strainer, and centrifuged for 2 minutes at 1000 g.

### Western blot

Tissues or cells were lysed in ice-cold Phosphosafe™ Extraction Reagent (EMD Millipore). Extracted protein concentrations were determined using a BCA™ Protein Assay (Fisher Scientific), and proteins were separated on 4–20% Mini-Protean TGX Stain-Free Gels (Bio-Rad). Protein gels were transferred to polyvinylidene fluoride (PVDF) membranes (Cytiva). Membranes were blocked with 5% nonfat milk (Bio-Rad) in TBST (0.1% Tween-20) and incubated overnight at 4°C with corresponding antibodies against high-mobility group nucleosome-binding domain-containing protein 3 (HMGN3, Bethyl Laboratories A301-191A), SET and MYND domain-containing 1 (SMYD1, Thermo Scientific PA5–84544), cysteine-aspartic acid protease 3 (caspase-3, Cell Signaling #9662s), cleaved caspase-3(Cell Signaling #9664s), histone H3 acetyl Lys27 (H3K27ac, Active Motif,#91193), and histone H3 lysine 4 mono-methylation (H3K4me1, Active Motif,#91415). The membrane was then washed three times with TBST and incubated with HRP-conjugated secondary antibodies (Cell signaling, #7074s, #7076s) at room temperature for 1 hour. Detection was performed using SuperSignal™ West Femto Maximum Sensitivity Substrate (Thermo Fisher Scientific). Membranes were developed and quantified by western blot densitometry using ImageJ software, with normalization to glyceraldehyde-3-phosphate dehydrogenase (GAPDH, Proteintech 60,004–1-lg) and expressed as a percentage of GAPDH.

### Quantitative RT-PCR analysis

Total RNA was extracted using TRIzol reagent and reverse-transcribed into cDNA using iScript™ Reverse Transcription Supermix for RT-PCR (Bio-Rad). qPCR was set up using SYBR Green Master Mix (Bio-Rad) and run on the QuantStudio Real-Time PCR System. All primers used are listed in Supplementary Table 2 Gene expression levels were quantified by the ^ΔΔ^Ct method using 18S as the housekeeping gene.

### Cell culture and siRNA transfection

Human cardiomyocyte-like AC16 cells were acquired from ATCC (CRL-3568) and cultured in DMEM/F-12 medium supplemented with 10% foetal bovine serum (FBS) and 100 U/mL penicillin (Sigma-Aldrich) at 37°C with 95% air and 5% CO₂ in a humidified incubator. The cells were typically cultured in 12-well plates at a density of 1 × 10^5^ cells. AC16 were transfected with siRNA: ON-TARGETplus SMARTpool Human HMGN3 (5 nmol, Horizon Discovery, L-011932-00–0005) or ON-TARGETplus Non-targeting Pool (5 nmol, Horizon Discovery, D-001810-10–05) at a concentration of 200 nM using Lipofectamine 2000 reagent (Thermo Scientific 11,668,019) according to the manufacturer’s instructions. Transfection efficiency was assessed after 48 or 72 hours.

### CCK-8 proliferation assay

AC16 cells were seeded at a density of 1.25 × 10^4^ cells per well in a 96-well cell culture plate (Fablab) overnight and transfected with HMGN3 or scrambled siRNA. After 72 hours, the culture medium was replaced, and 10 µL of CCK-8 reagent (DOJINDO Laboratories) was added to each well. Optical density (OD) at 450 nm was measured using a multifunction microplate reader following a 2-hour incubation at 37°C.

### Immunohistology

Pig cardiac tissue cryosections were fixed in 4% PFA for 20 minutes at room temperature, retrieved with IHC-Tek Epitope Retrieval Solution for 45 minutes in a steamer and blocked with 0.3% Triton X-100 and 10% donkey serum for 20 minutes at room temperature. Incubation with primary antibodies – rabbit anti-HMGN3 (Bethyl Laboratories A301-191A) and mouse anti-cardiac Troponin T (cTnT, Thermo ScientificC. The hearts were then perfused MA5–12960) – was performed overnight at 4°C. The samples were then stained with corresponding fluorescent secondary antibodies for 1 hour (Jackson ImmunoResearch Labs 715–545–150,711–605–152) and mounted with Antifade Mounting Medium containing DAPI (Vector Laboratories). Images were taken under Confocal Microscope (Confocal FV3000, Olympus).

### RNAseq

Three biological replicates, each consisting of approximately 1 million scramble- and siRNA-HMGN3–treated AC16 cells, were used in this study. Experimental procedures followed the protocol described in our previous publications [6,7,60]. Briefly, total RNA was extracted using the RNeasy Kit (Qiagen), and RNA quality was assessed with the Agilent 2100 Bioanalyzer. Ribosomal RNA was removed using the Illumina Ribo-Zero rRNA Removal Kit, and RNA-seq libraries were generated following standard protocols. Libraries were sequenced to a depth of at least 40 million paired-end reads (150 bp) per sample. Data analysis was performed in-house using custom pipelines. Sequencing reads were demultiplexed and aligned to the mm10 genome using STAR [61]. Differential expression analysis was performed with the DESeq() function from the DESeq2 [62] package using default parameters, yielding both raw and Benjamini – Hochberg adjusted p-values for each gene. Genes with an adjusted p-value < 0.05 and an absolute fold change ≥ 1.5 were defined as significantly differentially expressed. Gene Ontology analyses were conducted using ‘The Gene Ontology Resource’ with default parameters [63,64].

### ChIP-seq

H3K27ac ChIP was performed as described [65,66] with minor adaptations. Three biological replicates of ~ 1 × 10^6^ scramble- or siRNA-HMGN3–treated AC16 cells were crosslinked with 1% formaldehyde (10 min, RT) and quenched with 125 mM glycine. Cells were lysed and chromatin sonicated using the Pixul platform (Active Motif) for 60 min. Chromatin was incubated overnight at 4°C with Protein G/A Dynabeads (Invitrogen, #10003D) pre-bound to 1.5 µg H3K27ac antibody (Active Motif, #AB_2793305). Bead-bound complexes were washed and resuspended in TT buffer (10 mM Tris-HCl, pH 8.0, 0.05% Tween-20). Libraries were prepared on-bead with the NEBNext Ultra II Kit (NEB, #E7645L), PCR-amplified (14 cycles), barcoded with NEXTFLEX Unique Dual Index Barcodes, PCR amplified with Solexa 1 GA/1GB primers, and size-selected (200–500 bp) before sequencing on an Illumina NovaSeq SP100. ChIP-Seq analysis was performed as previously described [65,66]. H3K27ac peaks were analysed in the context of open chromatin using the annotatePeak.pl script from HOMER, with an ATAC-Seq peak set derived from a prior AC16 cell experiment [67]. Differential peak calling was done using HOMER’s getDiffExpression.pl function, with regions classified as differentially regulated if FDR < 0.05 and log₂ fold change > 0.58. Gene annotation was based on the nearest gene assigned by HOMER. Transcription factor motif enrichment was analysed with HOMER’s findMotifGenome.pl script, using differential regions (FDR < 0.05), and the -bg option for background regions.

## Results

### Analysis of human cardiac snRNA-seq data using the expression of chromatin structural factors can cluster healthy and disease populations in cardiomyocytes and fibroblasts

**Figure 1.**
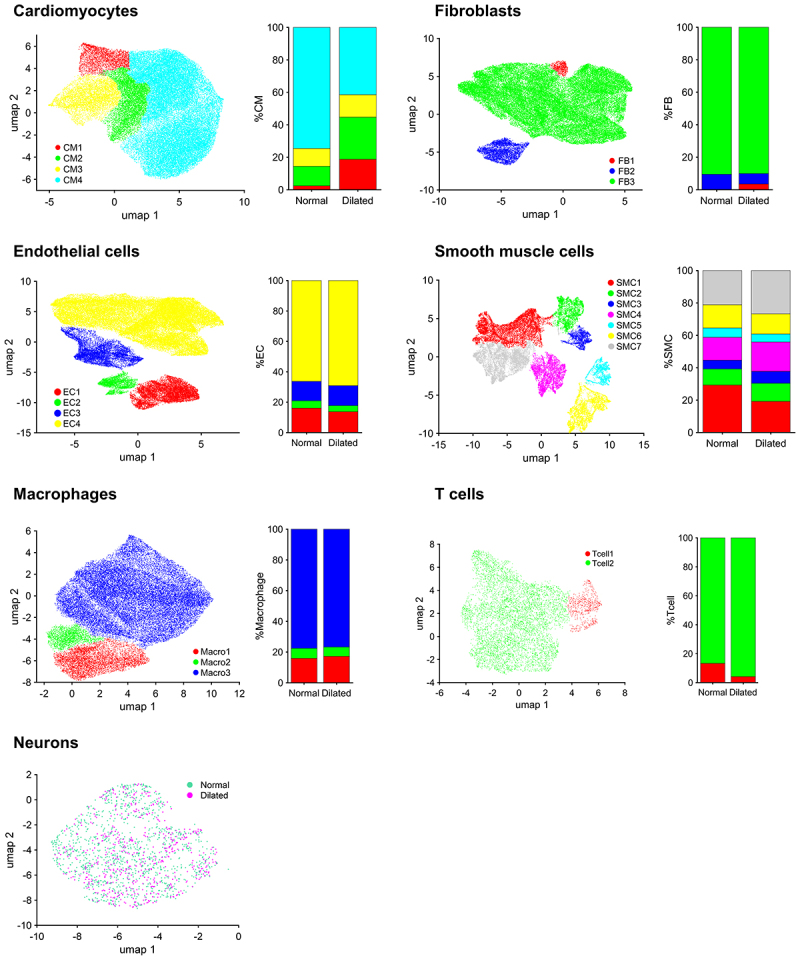
Unsupervised uniform manifold approximation and projection (UMAP). The different human cardiac cell types were clustered based on the expression of our curated list of chromatin structural factors. Each dot represents an individual cell, with its color indicating the corresponding cluster. Bar graphs display the percentage of each population relative to the total cell count within each respective cell type. The analysis successfully clustered all cell types, with the exception of neurons.

**Figure 2.**
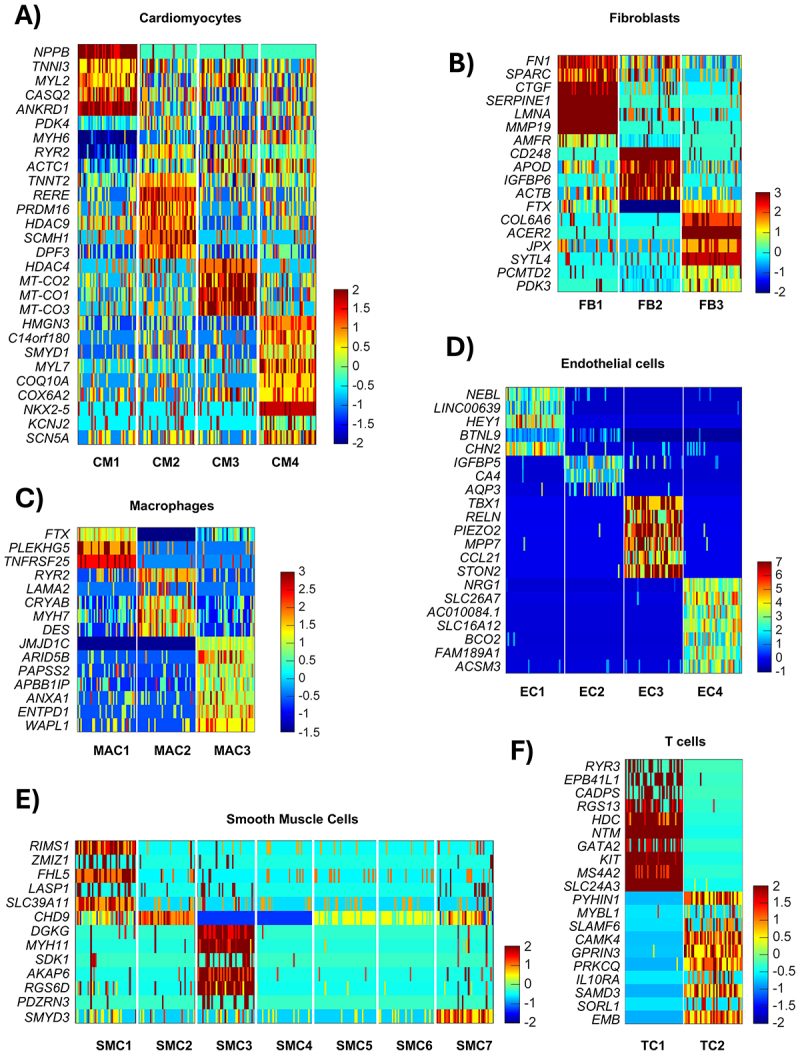
Clustering of human single-cell expression data reveals distinct pathological cardiomyocyte and fibroblast populations. Heatmaps displaying the expression (Log_2_ Fold change) of the characteristic marker genes for each identified cluster in the different cell types: cardiomyocytes (A), fibroblasts (B), macrophages (C), endothelial cells (D), smooth muscle cells (E), and T cells (F). Notably, CM1 shows downregulation of cardiac muscle contraction genes and upregulation of cardiomyopathy markers, while FB1 exhibits an increase in activation-related markers.

To build a comprehensive reference for studying the role of chromatin structural factors in the onset and progression of cardiac disease, we conducted an extensive literature review to refine the ‘Chromatin organization’ gene ontology term (GO:0006325) in the Mouse Genome Informatics database [68]. This effort compiled a table of 892 factors involved in chromatin remodelling and genomic structural topology (Supplementary Table 1). To test whether the generated list of structural factors could help identify healthy and sick cardiac cell subpopulations, we re-analysed the human-heart snRNA-seq dataset published by Dr. Lavine’s group [11]. Our analyses using conventional cell-specific markers reproduced the transcriptional differences observed between non-failing and dilated cardiomyopathy (DCM) donors across different heart cell types (Suppl. Figs. S1, 2). Applying our pipeline to analyse the data using the list of chromatin structural genes, we found that the transcriptional behaviour of these factors effectively clustered the different cell type populations, except for neurons (Figure 1 and Suppl. Fig. S4). Gene ontology analysis of each cluster revealed that, at least in cardiomyocytes and fibroblasts, our approach effectively distinguished between healthy and diseased phenotypes (Figure 2). In cardiomyocytes, the CM1 population, which increases from 3% in healthy hearts to 18% in DCM (Suppl. Fig. S4), was characterized by the downregulation of cardiac muscle contraction genes (*MYH6, RYR2, ACTC1, TNNT2*) and the upregulation of cardiomyopathy markers (*NPPB, TNNI3, MYL2, ANKRD1, CASQ2*). The expression of these genes gradually decreased in the other populations, with CM4 exhibiting a gene expression profile indicative of a healthy phenotype (Figure 2(A)). Similarly, in fibroblasts, activation-related markers (*FN1, CTCGF, SERPINE1, MMP19*) were highly expressed in the FB1 population, which is virtually absent in healthy hearts but appears in DCM (Suppl. Fig. S4). Expression of these markers decreases in the FB2 and FB3 populations, which are characterized by the expression of healthy markers (Figure 2(B)). In other cell types, although clustering was evident, gene expression differences between healthy and diseased phenotypes were less pronounced. In macrophages, we observed an increase in genes related to inflammatory and remodelling processes associated with macrophage activation (*FTX*, *PLEKHG5*, and *TNFRSF25*) (Figure 2(C)). A similar pattern was observed in endothelial cells, smooth muscle cells, and T cells, where gene expression changes involved pathways related to endothelial dysfunction and vascular remodelling (*NEBL*, *HEY1*, *CHN2*) (Figure 2(D)), the synthetic smooth muscle cell phenotype (*ZMIZ1*, *FHL5*) (Figure 2(E)), and immune responses or inflammation (*HDC*, *KIT*, *MS4A2*) (Figure 2(F)). However, these genes appear to play a less prominent role in defining pathological cell populations. Consequently, we focused our validation analyses on cardiomyocytes, where the phenotypic distinction between healthy and diseased clusters is more clearly defined.

**Figure 3.**
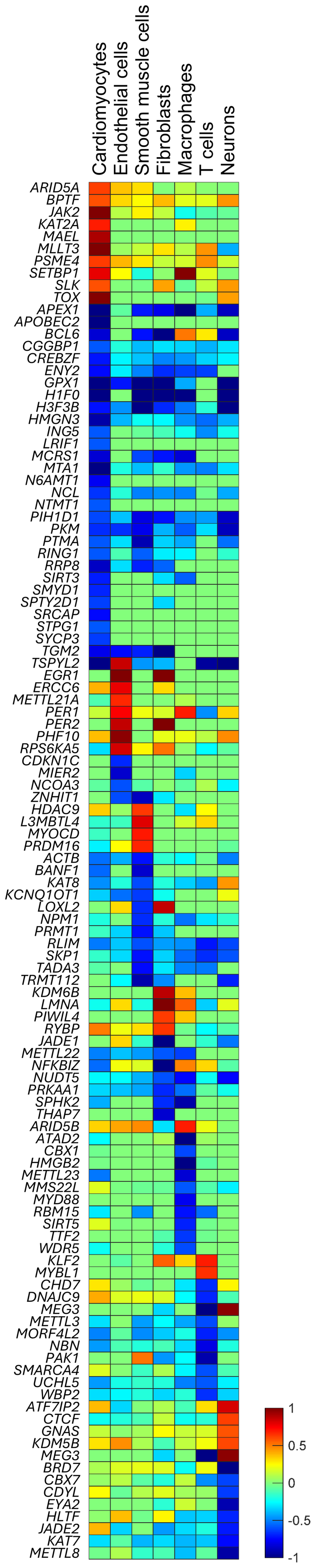
Cell type-specific expression of chromatin structural factors. Heatmap showing chromatin structural genes with significant differential expression in at least one cardiac cell type when comparing healthy donors and patients with dilated cardiomyopathy (DCM), ranked by log₂ fold-change. Most chromatin regulators exhibit distinct, cell type-specific expression patterns, suggesting that chromatin reorganization in heart failure is driven by different structural factors across cell populations. Red indicates genes upregulated in DCM relative to healthy samples, and blue indicates genes downregulated.

Upon analysing the expression of differentially expressed chromatin structural factors, we identified distinct transcriptional signatures for each cell type. Changes in the expression of factors such as *KAT2A, SRCAP, APOBEC2, LRIF1, N6AMT1*, and *SMYD1* were either specific to cardiomyocytes or more pronounced in this cell type compared to the others (Figure 3). In endothelial cells, we observed specific or more significant changes in the expression of factors such as *METTL21A, PER2, CDKN1C, MIER2*, and *NCOA3*. A similar pattern was evident in smooth muscle cells (e.g., *HDAC9, MYOCD, PRDM16, BANF1*, and *PRMT1*), fibroblasts (e.g., *KDM6B, PIWIL4, RYBP*, and *THAP7*), macrophages (e.g., *ARID5B, ATAD2, HMGB2, SIRT5, TTF2*, and *WDR5*), T-cells (e.g., *KLF2, MYBL1, METTL3, PAK1*, and *WBP2*), and neurons (e.g., *CTCF, KDM5D, BRD7, KAT7*, and *METTL8*). In contrast, chromatin structural factors such as *BPTF, APEX1, CCBPG1, CREGZF, H3F3B, HMGN3, MTA1, PKM, RLIM, NUDT5*, and *KAT7* exhibited differential expression across all cell types when comparing healthy and dilated cardiomyopathy conditions (Figure 3).

Notably, this analysis revealed that several members of well-established chromatin remodelling complexes were represented across the dataset. For instance, we identified multiple components of the NuRD/CoREST complex (*HDAC9, MTA1, MIER2, CHD7*, and *CDYL*), which couples histone deacetylation and chromatin remodelling to transcriptional repression. Similarly, factors from the SWI/SNF family (*SMARCA4, BRD7, SRCAP*, and *PHF10*), which regulate nucleosome positioning and accessibility, were also detected. We further identified components of the COMPASS/MLL-like and associated HAT complexes (*KDM6B, WDR5, KAT2A, KAT7, KAT8, ING5*, and *TADA3*), which regulate transcription through H3K27 demethylation and histone acetylation. Additionally, Polycomb group proteins (*RING1, CBX1, CBX7, RYBP*, and *L3MBTL4*), known for mediating transcriptional silencing via H2A ubiquitination and H3K27me3 recognition, were represented in our dataset. While our analysis was based on a curated list of chromatin-related genes, making the identification of these factors expected, the representation of multiple distinct complexes underscores the widespread impact of chromatin structural regulators across various epigenetic pathways.

**Figure 4.**
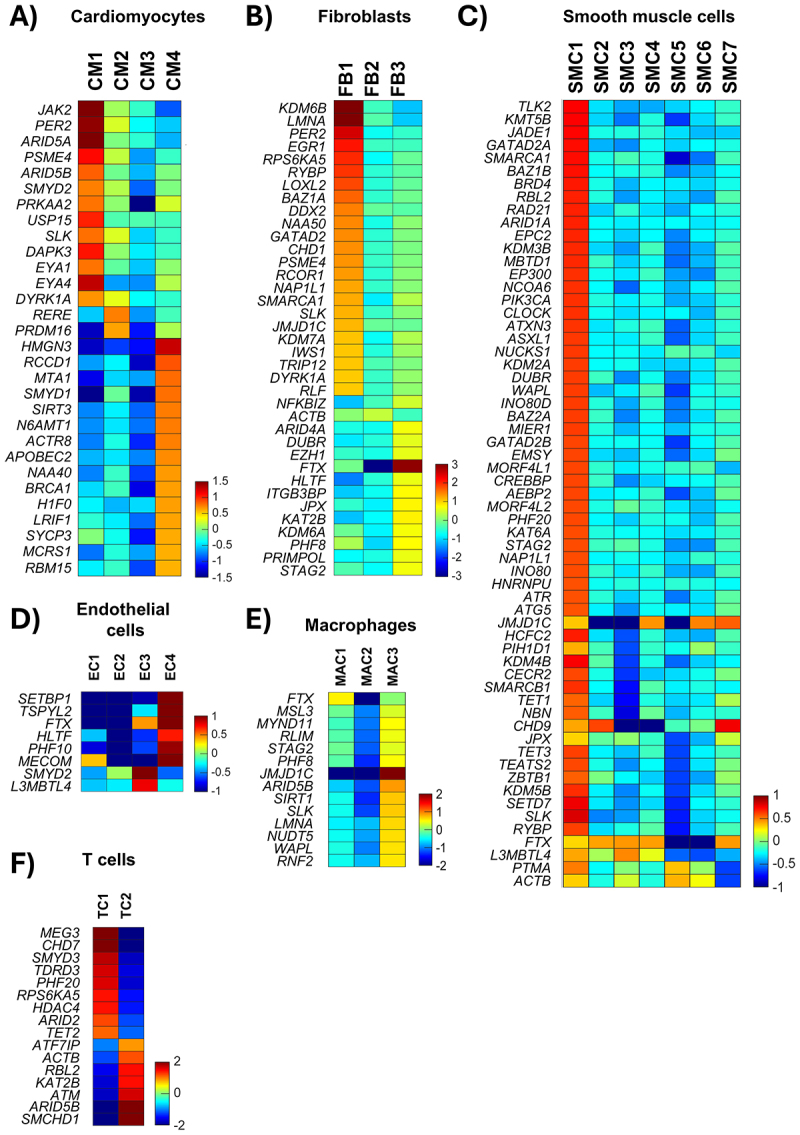
Cell cluster–specific expression of chromatin structural factors. Heatmaps depicting chromatin structural genes with significant differential expression between healthy donors and patients with dilated cardiomyopathy (DCM), ranked by log₂ fold-change within each cell cluster. Data are shown for cardiomyocytes (A), fibroblasts (B), smooth muscle cells (C), endothelial cells (D), macrophages (E), and T cells (F). For all graphs, red indicates genes upregulated in DCM relative to healthy samples, and blue indicates genes downregulated.

Further analysis of the different clusters within each cell type revealed distinct expression patterns of differentially expressed structural factors in cardiomyocytes and fibroblasts when comparing healthy and diseased populations. In cardiomyocytes, the CM1 population (diseased) showed a clear differential expression of factors such as *ARID5A, MTA1, HMGN3, SIRT3*, and *SMYD1* compared to CM4 (healthy) (Figure 4(A)). Similarly, in fibroblasts, the FB1 population (diseased) exhibited contrasting expression of factors like *KDM6B, LMNA, PER2, GATAD2*, and *SMARCA1D* compared to FB2 and FB3 (healthy) (Figure 4(B)). In other cell types, while we observed expression patterns of differentially expressed structural factors (Figure 4(C-F)), we were unable to distinctly separate diseased and healthy populations.

### Alterations in HMGN3 expression are linked to the pathological effects of dilated cardiomyopathy, pressure overload, and myocardial infarction

Since the role of the factor *HMGN3* in the cardiac phenotype has not been previously tested, we chose to focus on further studying its expression to validate the results of our snRNA transcriptional analyses. As a control, we also decided to examine the expression of the Histone Methyltransferase *SMYD1*, whose role in heart function is well-established and its expression is often downregulated in pathological cardiac conditions [69,70].

**Figure 5.**
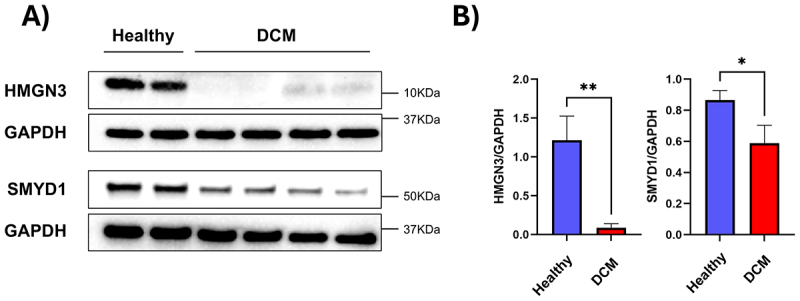
Validation of decreased *HMGN3* expression in cardiomyocytes from DCM patients. Western blot analysis (A) and corresponding quantification (B) confirm reduced levels of *HMGN3* and *SMYD1* in cardiomyocytes isolated from human hearts of patients with dilated cardiomyopathy (DCM), consistent with transcriptomic findings from scRNA-seq. Wb quantification was expressed as the mean ± s.D. Differences between groups were evaluated using an unpaired Student’s t-test. *p-value < 0.05, **p-value < 0.01.

**Figure 6.**
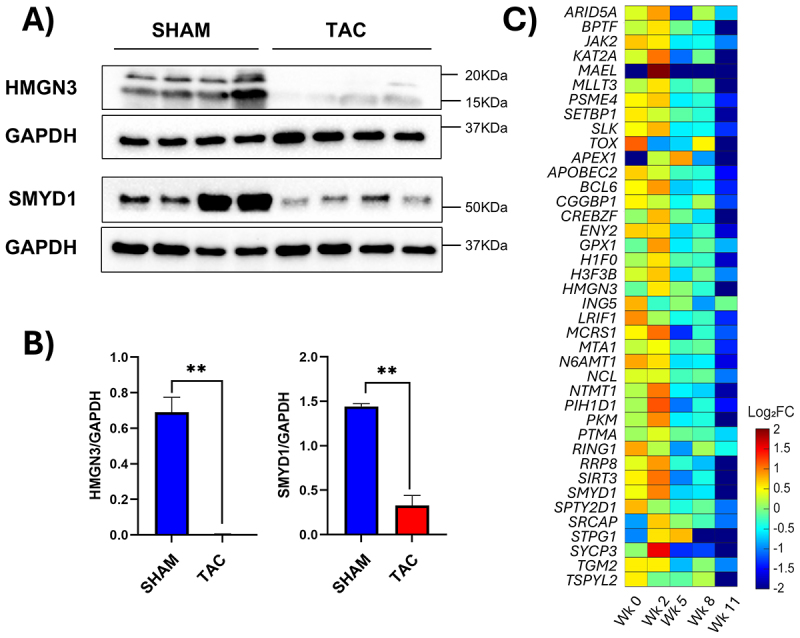
*HMGN3* levels decrease in response to induced pathological mouse hypertrophy. Western blot analysis (A) and corresponding quantification (B) show a significant decrease of *HMGN3* and *SMYD1* levels in isolated cardiomyocytes from TAC mice. (C) heatmap generated from RNA-seq data by Ren et al. (41) further confirms the decrease in *HMGN3* and *SMYD1* levels when comparing 11-week TAC mice to 0-week (pre-surgery) controls. Wb quantification was expressed as the mean ± s.D. Differences between groups were evaluated using an unpaired Student’s t-test. **p-value < 0.01. Red indicates genes upregulated in TAC relative to healthy samples, and blue indicates genes downregulated.

**Figure 7.**
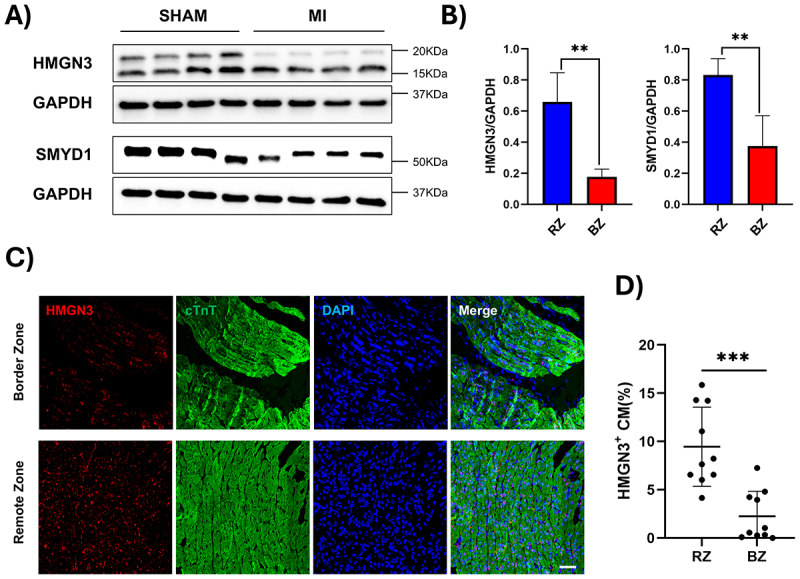
*HMGN3* levels decrease in response to induced myocardial infarction in pigs. Western blot analysis (A) and corresponding quantification (B) show a significant decrease of *HMGN3* and *Smyd1* levels in isolated cardiomyocytes from MI pig hearts. Immunofluorescence analyses (C) and corresponding quantification (D) demonstrate that *HMGN3* downregulation is more pronounced in the border zones of the myocardial infarcted areas than in regions of the heart distant from the lesion (remote zone). Wb quantification was expressed as the mean ± s.D. Differences between groups were evaluated using an unpaired Student’s t-test. **p-value < 0.01. IF quantification was expressed as the mean ± s.D. Differences between groups were evaluated using an unpaired Student’s t-test. ***p-value < 0.001.

To further validate our bioinformatic findings, we performed Western blot analysis on isolated cardiomyocytes (Suppl. Fig. S5) from both healthy individuals and donors with dilated cardiomyopathy, revealing a marked reduction in HMGN3 and SMYD1 protein levels (Figure 5(A, B)). Observed downregulation of SMYD1 also supports the diseased status of the used patients’ hearts [71,72] (Suppl. Fig. S6). To investigate whether the reduction in HMGN3 expression extends to other models of cardiac disease, we examined its expression in a mouse model of pressure overload-induced heart failure. In this model, transverse aortic constriction (TAC) was used to induce cardiac hypertrophy and subsequent heart failure (Suppl. Fig. S7). Western blot analysis confirmed significantly decreased levels of both HMGN3 and SMYD1 in TAC mice compared to SHAM-operated controls (Figure 6(A, B)), consistent with previous reports of SMYD1 downregulation in heart failure [73]. These results were further corroborated at the transcriptional level through bioinformatic reanalysis of a previously published bulk RNA-seq dataset comparing SHAM and TAC conditions at different time points after surgery [43] (Figure 6(C)). Finally, a decrease in *HMGN3* levels was also confirmed in a pig model of myocardial infarction. In this model, pigs underwent permanent ligation of the left anterior descending coronary artery, and cardiomyocytes were isolated 28 days post-intervention [44]. Compared to SHAM pigs, both *HMGN3* and *SMYD1* expression were significantly reduced in the myocardial infarcted group (Figure 7(A, B)). HMGN3 has two splice variants: HMGN3a (99 amino acids), the full-length, functionally active isoform, and HMGN3b (77 amino acids), a shorter variant lacking part of the C-terminal domain and showing reduced activity [74]. While these variants are not expressed in all cell types, they are present in pig cardiomyocytes. Importantly, in our experiments, the siRNA used primarily targets HMGN3a, the more active isoform. To assess whether the observed decrease in expression is present throughout the entire heart or localized to the border zone of the infarcted area, we performed immunofluorescence analyses. These studies revealed that the loss of *HMGN3* expression is most pronounced in the border zone of the infarcted area, compared to the remote (control) region (Figure 7(C, D)).

### HMGN3 depletion promotes cell death

**Figure 8.**
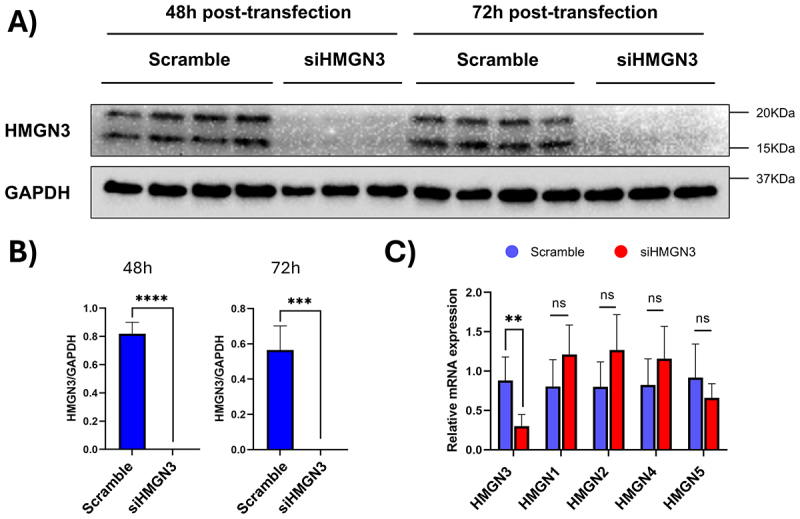
siRNA-mediated knockdown of *HMGN3* is effective and isoform-specific. Western blot analysis (A) and corresponding quantification (B) confirm the efficiency of *HMGN3* depletion cells when comparing AC16 cells transfected with scramble (control) and siRNA at 48 and 72 hours post-treatment. Wb quantification was expressed as the mean ± s.D. Differences between groups were evaluated using an unpaired Student’s t-test. ***p-value < 0.001. ****p-value < 0.0001. (C) qRT-PCR analysis of HMGN family member expression levels 72 hours post-transfection demonstrates the specificity of the used *HMGN3* siRNA. Differences between groups were assessed with an unpaired Student’s t-test. **p-value < 0.01.

**Figure 9.**
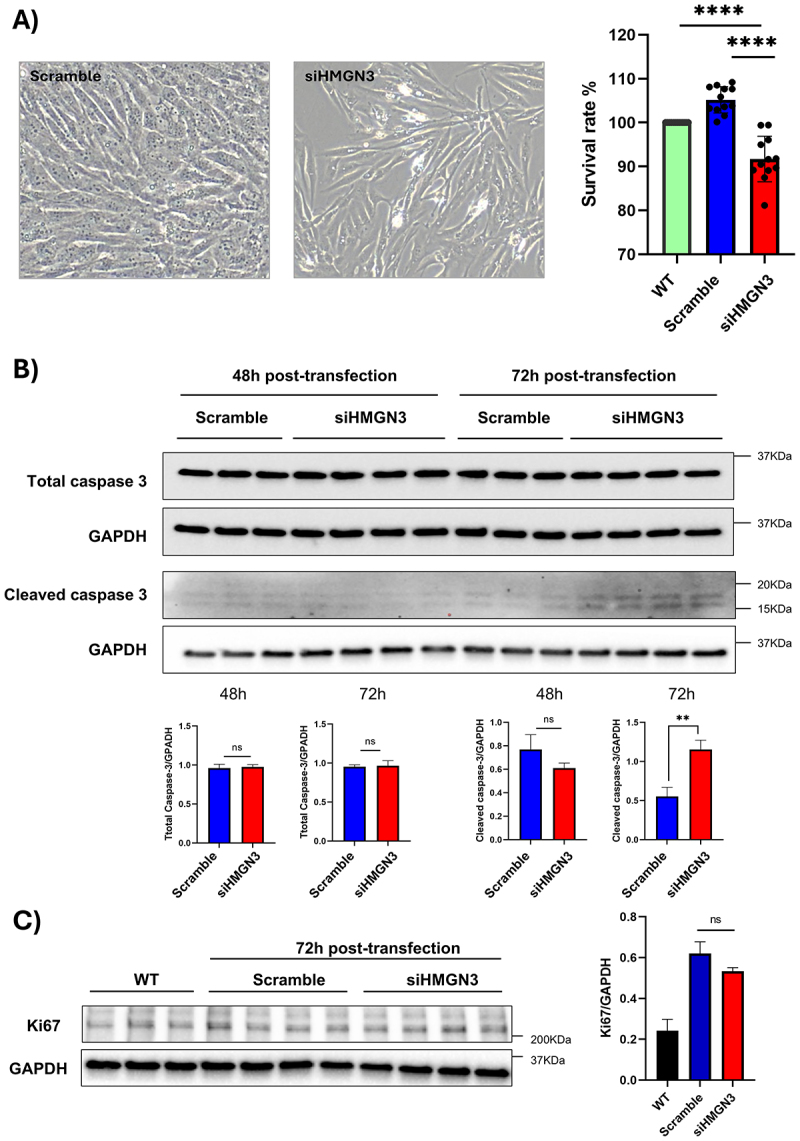
*HMGN3* knockdown promotes cell death and reduces cell proliferation. (A) visual inspection of AC16 cells transfected with scrambled or *HMGN3* siRNA (left) and survival rates 72 hours post-transfection (right) comparing non-transfected (WT), scrambled, and *HMGN3*-siRNA-transfected cells reveal an increase in cell death and a decrease in cell proliferation after *HMGN3* knockdown. Quantification was expressed as the mean ± s.D. And analyzed by one-way ANOVA followed by a post hoc Tukey test. ****p-value < 0.0001 (*n* = 12). (B) Western blot analysis (top) and corresponding quantifications (bottom) of caspase 3 and cleaved caspase 3 in AC16 cells transfected with scrambled (control) and HMGN3 siRNA at 48 and 72 hours post-transfection show no change in caspase 3 expression but an upregulation of the apoptosis marker, cleaved caspase 3. (C) Western blot analysis (left) and corresponding quantifications (right) of the Ki-67 in AC16 cells non transfected (WT) and transfected with scrambled (control) and HMGN3 siRNA at 72 hours post-transfection show no change in the proliferation marker levels. All wb quantifications are expressed as mean ± s.D., and differences between groups were assessed using an unpaired Student’s t-test. **p-value < 0.01.

Our bioinformatic analyses and western blot experiments demonstrated that *HMGN3* levels decrease in cardiomyocyte pathology. To investigate the role of *HMGN3* depletion in regulating the cardiac phenotype, we employed siRNA technology to knock down *HMGN3* expression in AC16 cells [75]. AC16 cells, a human cardiomyocyte-like cell line derived from ventricular heart tissue, proliferate while retaining key cardiac-specific markers, making them a suitable *in vitro* model for cardiac studies [76–78]. Our siRNA experiments revealed a significant decrease in *HMGN3* levels at 48- and 72-hours post-transfection (Figure 8(A, B)). RT-PCR confirmed that the knockdown specifically targeted HMGN3 without affecting other members of the HMGN family (Figure 8(C)). Cell counting at 72 hours post-transfection indicated increased cell death in the siHMGN3-transfected group compared to the scramble and wild-type groups (Figure 9(A)). To further validate the apoptotic response following HMGN3 depletion, we assessed total and cleaved caspase-3 levels. Western blotting revealed no change in total caspase-3 expression but a significant increase in the active form of caspase-3 at 72 hours post-transfection (Figure 9(B)). Additionally, to also evaluate the effect of HMGN3 depletion on cell proliferation, we performed Western blotting to compare Ki-67 levels (proliferation marker) in the scramble and siHMGN3 groups at 72 hours post-transfection. The data showed no significant differences (Figure 9(C)), suggesting that HMGN3 depletion does not impact cell proliferation, despite its role in promoting cell death.

### HMGN3 depletion induce changes in gene expression and reorganization of chromatin

Given that *HMGN3* primarily regulates nucleosomal positioning and chromatin accessibility is linked to cardiac phenotypes [79], we next examined the effect of *HMGN3* depletion on gene expression and chromatin organization.

**Figure 10.**
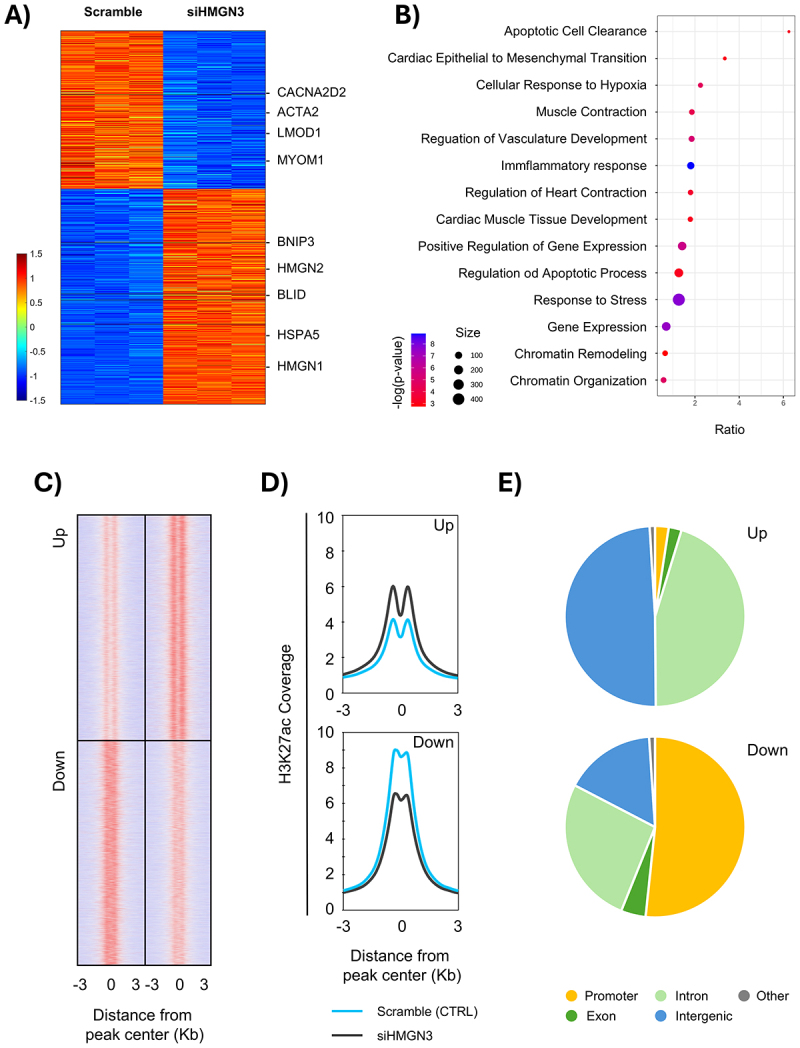
Loss of HMGN3 alters gene expression and chromatin regulation. (A) heatmap showing differentially expressed genes in scramble control versus siHMGN3 conditions. Key cardiac- and stress-related genes are highlighted on the right. (B) gene ontology (GO) enrichment analysis of differentially expressed genes upon HMGN3 knockdown. Dot size reflects gene set size, and color indicates adjusted p-value significance. Enriched categories include stress pathways, apoptosis, cardiac muscle development, and chromatin remodeling. (C) heatmaps of H3K27ac ChIP-seq signal centered on differential peaks (±3 kb from peak center) in siHMGN3 versus control conditions. Regions with increased (up) or decreased (down) acetylation are shown. (D) average profiles of H3K27ac occupancy across differential peaks in scramble (blue) and siHMGN3 (grey) cells, separated into upregulated (top) and downregulated (bottom) regions. The difference between scramble and siHMGN3 is more pronounced in regions losing H3K27ac signal (down) compared with those gaining the histone mark (up). (E) genomic distribution of differential H3K27ac peaks, classified as promoter, exon, intron, intergenic, or other, for both upregulated (top) and downregulated (bottom) sites. Peaks gaining H3K27ac signal are mainly located in regulatory regions (introns and intergenic regions), whereas those losing H3K27ac signal are enriched at promoters.

RNA-seq revealed 1484 genes upregulated and 1339 genes downregulated following HMGN3 depletion in AC16 cardiomyocytes (Figure 10(A) and Suppl. Fig. S8), arguing against a model in which HMGN3 functions solely as a unidirectional activator whose loss would simply cause global repression. Notably, among the induced transcripts we observed coordinated upregulation of chromatin-architecture and histone genes (e.g., HMGA1, HMGA2, ZMYND8, DPF3, H2BC7, H3C12, H4C16) consistent with compensatory changes in chromatin organization/remodelling that may enable activation of specific gene programmes despite reduced HMGN3.

Gene ontology analysis confirmed that HMGN3 depletion engages injury/defence programmes, including inflammatory response (e.g., *IL1A, IL1B, CXCL8, TLR4*), cellular response to hypoxia (e.g., *VEGFA, BNIP3, MIR210HG, CA9, CA12, LDHA, HK2, PDK1, SLC2A1*), and broader response to stress (e.g., *HSPA5, DDIT4, EIF4EBP2*), which together culminate in apoptosis – reflected by induction of *BNIP3, BLID, TP53I3, GLIPR1* and repression of survival/effector components such as *FAS, BBC3, BTG2* (Figure 10B). Concurrently, cardiac identity pathways are suppressed: muscle contraction and cardiac muscle tissue development decrease (e.g., *DES, ACTA2, LMOD1, TMOD1, OBSCN, MYOM1, PKP2*), as does regulation of heart contraction (e.g., *KCNJ11, KCNJ14, CACNA2D2*). In addition, induction of epithelial-to-mesenchymal transition (EMT) and extracellular matrix remodelling – marked by SNAI2 and matrix/adhesion effectors (e.g., *MMP1/2/3/9, PLOD2, LOXL1, ITGA2, ITGA3, CD44, CSPG4, LAMB3, LAMC2, THBS1, TNC*) – further destabilizes cardiac lineage fidelity by promoting dedifferentiation and tissue remodelling, processes more characteristic of pathological remodelling than of stable cardiomyocyte identity (Figure 10(B)). Collectively, our RNA-seq data suggest that HMGN3 depletion reshapes the regulatory landscape of AC16 cells in a manner consistent with loss of cardiomyocyte-like programmes and induction of maladaptive remodelling pathways.

To assess how HMGN3 shapes regulatory chromatin, we profiled H3K27ac by ChIP – seq in AC16 cells before and after HMGN3 depletion. Heatmaps centered on differentially regulated regions revealed a genome-wide redistribution of H3K27ac: 8,148 regions gained and 8,299 regions lost signal upon depletion (Figure 10(C)), mirroring the two-directional transcriptional response in our RNA-seq data. Regions that gained H3K27ac displayed a bimodal profile, consistent with acetylation of nucleosomes flanking, indicative of newly accessible chromatin and often observed at active enhancers. In contrast, regions that lost H3K27ac exhibited a sharp, unimodal peak characteristic of focused acetylation at established regulatory sites such as strong promoters. Notably, the magnitude of loss at downregulated regions exceeded the magnitude of gain at newly acetylated regions, revealing an asymmetry after HMGN3 depletion that favours repression of previously acetylated sites (Figure 10(D)). Consistent with this imbalance, bulk H3K27ac levels measured by Western blot (normalized to total H3) decreased in AC16 upon HMGN3 depletion (Suppl. Fig. S9A).

Motif analysis of differential H3K27ac regions revealed distinct transcription factor programmes associated with loss versus gain of acetylation following HMGN3 depletion (Suppl. Fig. S9B). Regions losing H3K27ac exhibited a motif signature typical of promoter-proximal, stably active genomic sites—*YY1*^*80*^, *NF-Y*^*81*^, *ETS/ETV7*^*82*^, *ATF*^*83*^, and diverse ZNFs. By contrast, regions gaining H3K27ac upon HMGN3 loss showed a signature consistent with distal, stimulus-responsive regulatory programmes, including *AP-1*^*84*^, *TEAD1*^*85*^, *GLIS2*^*86*^, and *BNC2*^*87*^. Genomic annotation of the differential H3K27ac peaks further supported these patterns: regions losing acetylation were predominantly at promoters, whereas regions gaining acetylation were largely intronic or intergenic (Figure 10(E)).

Integrating ChIP – seq with RNA-seq further reinforced this model. Downregulated genes were preferentially linked to promoter-proximal peaks that lose H3K27ac, whereas upregulated genes were associated with peaks gaining H3K27ac in intronic and intergenic regions (Suppl. Fig. S9C). Together, these data indicate that HMGN3 maintains focused H3K27ac at promoter-like regions while restraining the emergence of new, distal acetylated sites, producing a depletion phenotype quantitatively skewed towards loss at established regulatory elements.

## Discussion

The spatial organization of the genome within the nucleus plays a critical role in regulating gene expression at both local and global levels of chromatin structure. Our previous Hi-C and ATAC-seq analyses have linked alterations in higher-order chromatin structure and accessibility to the progression of cardiac pathology [6,7]. We have also shown that histone H1—the linker histone responsible for nucleosome compaction – acts as a key mediator between cellular mechanics and chromatin remodelling during fibroblast activation, with a notable effect on cardiac fibrosis [88]. Together, these findings underscore the importance of chromatin architecture in disease progression.

To identify the upstream factors driving these structural changes, we re-analysed a publicly available single-cell RNA-seq dataset comparing healthy human donors and patients with dilated cardiomyopathy (DCM). This analysis only focused on the differential expression of a curated panel of chromatin structural regulators and was able to resolve all major cardiac cell types. We also identified a distinct diseased cardiomyocyte population along with an activated fibroblast cluster, suggesting that changes in the expression of chromatin-structuring factors have a direct impact on establishing pathological cell states within the heart and, therefore, the cardiac phenotype. Our study observed disease-associated markers in additional cell types, we were only able to establish clear associations between chromatin regulator expression and pathological phenotypes in cardiomyocytes and fibroblasts. This likely reflects the fact that these two cell populations are particularly vulnerable during the progression of DCM.

Comparative analysis of structural factor expression between healthy and DCM samples revealed that each cardiac cell type displays a distinct regulatory signature. This finding suggests that cardiac chromatin remodelling is driven by cell – type – specific factors. A similar trend was observed when examining subclusters within individual cell types, further supporting the idea that chromatin-mediated aspects of cellular identity are shaped by different structural regulators across cell populations.

Our study identified several key factors involved in regulating the cardiac phenotype through chromatin remodelling, some of which are cell-specific, while others act across multiple cell types. In this investigation, we focused on *HMGN3*, a factor never studied in the heart before and a member of the HMGN family, whose primary role is to modulate chromatin accessibility by regulating the association of histone H1 with the nucleosome [16]. Our data show a significant reduction in HMGN3 expression in DCM patients across all cell types, with the most notable differences observed in cardiomyocytes. These transcriptional findings were validated at the protein level through Western blot analysis of isolated cardiomyocytes from DCM patients, as well as in mouse TAC and pig MI models. In myocardial infarction, we also observed a reduction in HMGN3 levels in the border zone of the infarcted areas – a dynamic region undergoing a combination of injury, repair, and remodelling. These results suggest that chromatin structure regulation mediated by HMGN3 May play a crucial role in inflammation and repair processes, including tissue regeneration, and further support its involvement in fibroblast activation and fibrosis promotion in this area.

This study also demonstrated that HMGN3 knockdown promotes cell death in AC16 cells, a human cardiomyocyte cell line widely used to investigate cardiac function and disease mechanisms. Our RNA-seq analysis suggests that this effect is likely driven by the activation of multiple stress pathways that promote apoptosis. In support of its relevance to disease, our western blotting also revealed reduced HMGN3 levels in TAC hearts and DCM patient samples, indicating that diminished HMGN3 May represent an upstream chromatin perturbation associated with pathological remodelling and cardiomyocyte loss in heart failure. Our RNA-seq data further show that HMGN3 depletion triggers a bimodal transcriptional response, in which the loss of HMGN3 alters chromatin architecture, leading to both gene activation and silencing. Gene ontology analysis supports this finding by revealing that HMGN3 depletion not just causes a massive loss of chromatin accessibility and widespread gene downregulation, but also highlights that, in addition to terms related to apoptosis and cardiac phenotypes, HMGN3 regulates chromatin architecture and histone genes involved in chromatin remodelling and organization, which may facilitate gene activation.

ChIP-seq analysis of the active histone mark H3K27ac, a proxy for chromatin accessibility, revealed a bimodal chromatin reorganization following HMGN3 depletion, consistent with the RNA-seq data. Additionally, genomic annotation and motif analysis of differential H3K27ac peaks after HMGN3 depletion showed that while peaks losing H3K27ac are predominantly located in promoters, upregulated peaks are primarily found in intergenic regions. A similar trend was observed when focusing on genes differentially expressed after HMGN3 depletion, suggesting that the first group of peaks regulates gene expression through direct promoter modification, while the second group mediates gene expression via regulatory elements. Together, these data indicate that HMGN3 depletion triggers a complex chromatin reorganization that facilitates both gene activation and repression, contributing to the observed shifts in gene expression and cellular behaviour.

In conclusion, our study has: (1) confirmed that the expression of chromatin structural factors distinguishes healthy and diseased phenotypes in cardiomyocytes and fibroblasts; (2) provided a list of potential effectors involved in chromatin structural changes associated with heart disease; (3) demonstrated a decrease in HMGN3 levels during heart pathology; and (4) identified HMGN3 as a key mediator of cardiac cell survival by regulating survival genes and cardiac phenotypic pathways through modulation of chromatin structure. Despite these valuable insights, we acknowledge that the limited availability of high-quality cardiac sncRNA-seq datasets across diverse cardiac diseases restricts our ability to determine whether our list of structural factors can also identify pathological cell populations beyond cardiomyocytes and fibroblasts, such as endothelial or smooth muscle cells. While this represents an important limitation, we anticipate that future studies will address these gaps allowing us to further refine our understanding of chromatin remodelling in different cardiac diseases through studies building on our approach.

## Supplementary Material

Supplemental Material

Supplemental Material

## Data Availability

The human and mouse datasets previously published and used to generate the findings of this study are openly available in the Gene Expression Omnibus, with reference numbers GSE183852 (https://www.ncbi.nlm.nih.gov/geo/query/acc.cgi?acc=GSE183852) and GSE120064 (https://www.ncbi.nlm.nih.gov/geo/query/acc.cgi), respectively. RNA-seq and ChIP-seq data specifically generated for this project are also available in the Gene Expression Omnibus under the reference number GSE306626 (https://www.ncbi.nlm.nih.gov/geo/query/acc.cgi?acc = GSE306626). The rest of the generated data are freely accessible through the Zenodo Data repository (https://zenodo.org/records/17035888).
