# Supplementary material for: Chromatin structural gene expression stratifies cardiac cell populations in health and disease
Source: Epigenetics. 2025 Oct 21;20(1):2566505. doi: 10.1080/15592294.2025.2566505 (PMC12542603; doi:10.1080/15592294.2025.2566505)
Supplement: Supplemental Material [file KEPI_A_2566505_SM2805.zip › SI-tables/Table_1.docx]

| **MGI Gene ID** | **Symbol** | **Name** | **Chr** | **MGI Ref. ID / PubMed ID** |
| --- | --- | --- | --- | --- |
| MGI:87853 | a | nonagouti | 2 | J:82396 / PMID:12601169 |
| MGI:1917931 | Abraxas1 | BRCA1 A complex subunit | 5 | J:60000 |
| MGI:87904 | Actb | actin, beta | 5 | J:283189 |
| MGI:1861453 | Actl6a | actin-like 6A | 3 | J:161428 / PMID:9845365 |
| MGI:1933548 | Actl6b | actin-like 6B | 5 | J:80453 / PMID:12437990 |
| MGI:1924748 | Actr5 | ARP5 actin-related protein 5 | 2 | J:164563 |
| MGI:1860775 | Actr8 | ARP8 actin-related protein 8 | 14 | J:342607 |
| MGI:1338038 | Aebp2 | AE binding protein 2 | 6 | J:60000 |
| MGI:1342279 | Aicda | activation-induced cytidine deaminase | 6 | J:164563 |
| MGI:1353471 | Airn | antisense Igf2r RNA | 17 | J:77594 / PMID:11845212 / PMID:18988810 |
| MGI:2384034 | Alkbh1 | alkB homolog 1, histone H2A dioxygenase | 12 | J:276409 |
| MGI:1919291 | Alkbh4 | alkB homolog 4, lysine demethylase | 5 | J:278437 |
| MGI:1914878 | Anp32b | acidic nuclear phosphoprotein 32 family member B | 4 | J:342605 |
| MGI:1913721 | Anp32e | acidic nuclear phosphoprotein 32 family member E | 3 | J:60000 |
| MGI:2384888 | Antkmt | adenine nucleotide translocase lysine methyltransferase | 17 | J:346132 |
| MGI:107765 | Apbb1 | amyloid beta precursor protein binding family B member 1 | 7 | J:155856 |
| MGI:88042 | Apex1 | apurinic/apyrimidinic endonuclease 1 | 14 | J:164563 |
| MGI:1919353 | Aplf | aprataxin and PNKP like factor | 6 | J:164563 |
| MGI:103298 | Apobec1 | apolipoprotein B mRNA editing enzyme, catalytic polypeptide 1 | 6 | J:175307 |
| MGI:1343178 | Apobec2 | apolipoprotein B mRNA editing enzyme, catalytic polypeptide 2 | 17 | J:164563 |
| MGI:88059 | App | amyloid beta precursor protein | 16 | J:164563 |
| MGI:1915925 | Arb2a | ARB2 cotranscriptional regulator A | 13 | J:161428 |
| MGI:1935147 | Arid1a | AT-rich interaction domain 1A | 4 | J:69532 / PMID:11318604 / PMID:18448678 |
| MGI:1926129 | Arid1b | AT-rich interaction domain 1B | 17 | J:199644 / PMID:23785148 |
| MGI:1924294 | Arid2 | AT-rich interaction domain 2 | 15 | J:320039 |
| MGI:1328360 | Arid3a | AT-rich interaction domain 3A | 10 | PMID: 34663594 |
| MGI:1930768 | Arid3b | AT-rich interaction domain 3B | 9 | PMID: 27512077 |
| MGI:3650624 | Arid3c | AT-rich interaction domain 3C | 4 | PMID: 21955986 |
| MGI:2444354 | Arid4a | AT-rich interaction domain 4A | 12 | J:113402 / PMID:17043311 / PMID:18728284 / PMID:17043311 |
| MGI:2137512 | Arid4b | AT-rich interaction domain 4B | 13 | J:113402 / PMID:17043311 |
| MGI:2443039 | Arid5a | AT-rich interaction domain 5A | 1 | PMID: 38866324 |
| MGI:2175912 | Arid5b | AT-rich interaction domain 5B | 10 | PMID: 38224186 |
| MGI:1913653 | Asf1a | anti-silencing function 1A histone chaperone | 10 | J:164563 |
| MGI:1914179 | Asf1b | anti-silencing function 1B histone chaperone | 8 | J:85416 / PMID:12842904 |
| MGI:2183158 | Ash1l | ASH1 like histone lysine methyltransferase | 3 | J:346132 |
| MGI:1344416 | Ash2l | ASH2 like histone lysine methyltransferase complex subunit | 8 | J:164563 |
| MGI:2684063 | Asxl1 | ASXL transcriptional regulator 1 | 2 | J:60000 |
| MGI:1917722 | Atad2 | ATPase family | 15 | J:161428 |
| MGI:2444798 | Atad2b | ATPase family | 12 | J:161428 |
| MGI:109349 | Atf2 | activating transcription factor 2 | 2 | J:342605 |
| MGI:1858965 | Atf7ip | activating transcription factor 7 interacting protein | 6 | J:161428 |
| MGI:1922579 | Atf7ip2 | activating transcription factor 7 interacting protein 2 | 16 | J:161428 |
| MGI:1277186 | Atg5 | autophagy related 5 | 10 | J:204746 / PMID:23863932 |
| MGI:1921494 | Atg7 | autophagy related 7 | 6 | J:204746 / PMID:23863932 |
| MGI:107202 | Atm | ataxia telangiectasia mutated | 9 | J:346132 |
| MGI:1915323 | Atpsckmt | ATP synthase C subunit lysine Nmethyltransferase | 15 | J:346132 |
| MGI:108028 | Atr | ataxia telangiectasia and Rad3 related | 9 | J:346132 |
| MGI:103067 | Atrx | ATRX | X | J:164563 |
| MGI:1099442 | Atxn3 | ataxin 3 | 12 | J:346132 |
| MGI:3036270 | Atxn7l3 | ataxin 7-like 3 | 11 | J:60000 |
| MGI:894678 | Aurka | aurora kinase A | 2 | J:346132 |
| MGI:107168 | Aurkb | aurora kinase B | 11 | J:346132 |
| MGI:1096327 | Axin1 | axin 1 | 17 | J:82396 / PMID:12601169 |
| MGI:1915501 | Babam1 | BRISC and BRCA1 A complex member 1 | 8 | J:164563 |
| MGI:1333875 | Babam2 | BRISC and BRCA1 A complex member 2 | 5 | J:60000 |
| MGI:1919439 | Bag6 | BCL2-associated athanogene 6 | 17 | J:60000 |
| MGI:2679272 | Bahcc1 | BAH domain and coiled-coil containing 1 | 11 | J:301382 / PMID:32969152 |
| MGI:2139371 | Bahd1 | bromo adjacent homology domain containing 1 | 2 | J:161428 |
| MGI:1346330 | Banf1 | BAF nuclear assembly factor 1 | 19 | J:164563 |
| MGI:1889023 | Banp | BTG3 associated nuclear protein | 8 | J:60000 |
| MGI:1206586 | Bap1 | Brca1 associated protein 1 | 14 | J:161428 |
| MGI:1309478 | Baz1a | bromodomain adjacent to zinc finger domain 1A | 12 | J:76488 / PMID:11980720 |
| MGI:1353499 | Baz1b | bromodomain adjacent to zinc finger domain | 5 | J:76488 / PMID:11980720 |
| MGI:2151152 | Baz2a | bromodomain adjacent to zinc finger domain | 10 | J:71381 / PMID:11532953 / PMID:16678107 / PMID:20168299 / PMID:16678107 / PMID:20168299 |
| MGI:2136782 | BC004004 | cDNA sequence BC004004 | 17 | J:164563 |
| MGI:107187 | Bcl6 | B cell leukemia/lymphoma 6 | 16 | J:197487 / PMID:23160044 |
| MGI:1924295 | Bcl7a | B cell CLL/lymphoma 7A | 5 | J:283189 |
| MGI:1332238 | Bcl7b | B cell CLL/lymphoma 7B | 5 | J:283189 |
| MGI:1332237 | Bcl7c | B cell CLL/lymphoma 7C | 7 | J:283189 |
| MGI:1918708 | Bcor | BCL6 interacting corepressor | X | J:164563 |
| MGI:2443910 | Bcorl1 | BCL6 co-repressor-like 1 | X | J:60000 |
| MGI:3705232 | Bend2 | BEN domain containing 2 | X | J:325496 / PMID:35613276 |
| MGI:2677212 | Bend3 | BEN domain containing 3 | 10 | J:164563 |
| MGI:2154263 | Bicra | BRD4 interacting chromatin remodeling complex associated protein | 7 | J:283189 |
| MGI:2673855 | Bicral | BRD4 interacting chromatin remodeling complex associated protein like | 17 | J:283189 |
| MGI:88174 | Bmi1 | Bmi1 polycomb ring finger oncogene | 2 | J:104662 / PMID:16359901 / PMID:16687444 / PMID:24105743 |
| MGI:5590788 | Bmncr | bone marrow associated noncoding RNA | 1 | J:270992 / PMID:30352426 |
| MGI:88184 | Bmyc | brain expressed myelocytomatosis oncogene | 2 | J:164563 |
| MGI:2444008 | Bptf | bromodomain PHD finger transcription factor | 11 | J:164563 |
| MGI:104537 | Brca1 | breast cancer 1 | 11 | J:79872 / PMID:12419249 |
| MGI:109337 | Brca2 | breast cancer 2, early onset | 5 | J:346132 |
| MGI:2389572 | Brcc3 | BRCA1/BRCA2-containing complex | X | J:164563 |
| MGI:3647286 | Brcc3dc | BRCA1/BRCA2-containing complex | 10 | J:164563 |
| MGI:1924161 | Brd1 | bromodomain containing 1 | 15 | J:346132 |
| MGI:99495 | Brd2 | bromodomain containing 2 | 17 | J:164563 |
| MGI:1914632 | Brd3 | bromodomain containing 3 | 2 | J:161428 |
| MGI:1888520 | Brd4 | bromodomain containing 4 | 17 | J:161428 |
| MGI:1349766 | Brd7 | bromodomain containing 7 | 8 | J:164563 |
| MGI:1925906 | Brd8 | bromodomain containing 8 | 18 | J:60000 |
| MGI:2145317 | Brd9 | bromodomain containing 9 | 13 | J:283189 |
| MGI:1891374 | Brdt | bromodomain | 5 | J:84555 / PMID:12861021 |
| MGI:1926033 | Brpf1 | bromodomain and PHD finger containing, 1 | 6 | J:240552 |
| MGI:2146836 | Brpf3 | bromodomain and PHD finger containing, 3 | 17 | J:346132 |
| MGI:1100510 | Bub1 | BUB1, mitotic checkpoint serine/threonine kinase | 2 | J:346132 |
| MGI:1913388 | Bud23 | BUD23, rRNA methyltransferase and ribosome maturation factor | 5 | J:60000 |
| MGI:1913208 | Carm1 | coactivator-associated arginine methyltransferase 1 | 9 | J:161428 |
| MGI:105369 | Cbx1 | chromobox 1 | 11 | J:161428 |
| MGI:88289 | Cbx2 | chromobox 2 | 11 | J:60000 |
| MGI:108515 | Cbx3 | chromobox 3 | 6 | J:161428 |
| MGI:1195985 | Cbx4 | chromobox 4 | 11 | J:60000 |
| MGI:109372 | Cbx5 | chromobox 5 | 15 | J:161428 |
| MGI:3512628 | Cbx6 | chromobox 6 | 15 | J:60000 |
| MGI:1196439 | Cbx7 | chromobox 7 | 15 | J:60000 |
| MGI:1353589 | Cbx8 | chromobox 8 | 11 | J:60239 / PMID:10721694 / PMID: 3059706 |
| MGI:1916218 | Cdan1 | codanin 1 | 2 | J:161428 |
| MGI:1915099 | Cdca5 | cell division cycle associated 5 | 19 | PMID: 31205541 |
| MGI:88351 | Cdk1 | cyclin dependent kinase 1 | 10 | J:346132 |
| MGI:104772 | Cdk2 | cyclin dependent kinase 2 | 10 | J:164563 |
| MGI:101765 | Cdk5 | cyclin dependent kinase 5 | 5 | J:346132 |
| MGI:1328368 | Cdk9 | cyclin dependent kinase 9 | 2 | J:164563 |
| MGI:104564 | Cdkn1c | cyclin dependent kinase inhibitor 1C | 7 | J:147145 / PMID:19276117 |
| MGI:1339956 | Cdyl | chromodomain protein, Y chromosome-like | 13 | J:206754 |
| MGI:104982 | Cebpg | CCAAT/enhancer binding protein gamma | 7 | J:113689 |
| MGI:1923799 | Cecr2 | CECR2, histone acetyl-lysine reader | 6 | J:342607 |
| MGI:88375 | Cenpa | centromere protein A | 5 | J:164563 |
| MGI:2147897 | Cenpi | centromere protein I | X | J:161428 |
| MGI:1919405 | Cenpn | centromere protein N | 8 | J:72247 |
| MGI:1913586 | Cenpp | centromere protein P | 13 | J:72247 |
| MGI:1920389 | Cenpv | centromere protein V | 11 | J:161428 |
| MGI:1344403 | Cfdp1 | craniofacial development protein 1 | 8 | J:161428 |
| MGI:2146370 | Cggbp1 | CGG triplet repeat binding protein 1 | 16 | J:164563 |
| MGI:5624990 | Chaer1 | cardiac hypertrophy associated epigenetic regulator 1 | 5 | J:240516 / PMID:27618650 |
| MGI:1351331 | Chaf1a | chromatin assembly factor 1 | 17 | J:164563 |
| MGI:1314881 | Chaf1b | chromatin assembly factor 1 | 16 | J:164563 |
| MGI:88393 | Chd1 | chromodomain helicase DNA binding protein 1 | 17 | J:105708 / PMID:16415155 |
| MGI:1915308 | Chd1l | chromodomain helicase DNA binding protein 1-like | 3 | J:161428 |
| MGI:2448567 | Chd2 | chromodomain helicase DNA binding protein 2 | 7 | J:161428 |
| MGI:1344395 | Chd3 | chromodomain helicase DNA binding protein 3 | 11 | J:54004 / PMID:10204490 |
| MGI:1344380 | Chd4 | chromodomain helicase DNA binding protein 4 | 6 | J:161428 |
| MGI:3036258 | Chd5 | chromodomain helicase DNA binding protein 5 | 4 | J:161428 |
| MGI:1918639 | Chd6 | chromodomain helicase DNA binding protein 6 | 2 | J:161428 |
| MGI:2444748 | Chd7 | chromodomain helicase DNA binding protein 7 | 4 | J:161428 |
| MGI:1915022 | Chd8 | chromodomain helicase DNA binding protein 8 | 14 | J:161428 |
| MGI:1924001 | Chd9 | chromodomain helicase DNA binding protein 9 | 8 | J:346132 |
| MGI:1202065 | Chek1 | checkpoint kinase 1 | 9 | J:177150 |
| MGI:2135796 | Chrac1 | chromatin accessibility complex 1 | 15 | J:161428 |
| MGI:1913761 | Chtop | chromatin target of PRMT1 | 3 | J:161428 |
| MGI:99698 | Clock | clock circadian regulator | 5 | J:346132 |
| MGI:1913673 | Coprs | coordinator of PRMT5 | 8 | J:164563 |
| MGI:1098280 | Crebbp | CREB binding protein | 16 | J:346132 |
| MGI:2675296 | Crebzf | CREB/ATF bZIP transcription factor | 7 | J:342587 |
| MGI:109447 | Ctcf | CCCTC-binding factor | 8 | J:164563 / PMID: 19563753 |
| MGI:3652571 | Ctcfl | CCCTC-binding factor like | 2 | J:164563 / PMID: 38297316 |
| MGI:109345 | Ctr9 | CTR9 homolog | 7 | J:203796 / PMID:24036311 |
| MGI:1203520 | Dapk3 | death-associated protein kinase 3 | 10 | J:60000 |
| MGI:1197015 | Daxx | Fas death domain-associated protein | 17 | J:164563 |
| MGI:2445220 | Dcaf1 | DDB1 and CUL4 associated factor 1 | 9 | J:346132 |
| MGI:2684929 | Dcaf13 | DDB1 and CUL4 associated factor 13 | 15 | J:164563 |
| MGI:1202384 | Ddb1 | damage specific DNA binding protein 1 | 19 | J:164563 |
| MGI:2443590 | Ddx11 | DEAD/H box helicase 11 | 17 | J:342587 |
| MGI:1860494 | Ddx21 | DExD box helicase 21 | 10 | J:164563 |
| MGI:1921601 | Ddx23 | DEAD box helicase 23 | 15 | J:164563 |
| MGI:102670 | Ddx4 | DEAD box helicase 4 | 13 | J:243488 |
| MGI:1926209 | Dek | DEK proto-oncogene | 13 | J:320057 |
| MGI:2177178 | Dicer1 | dicer 1 | 12 | J:96031 / PMID:15713842 |
| MGI:1913483 | Dmap1 | DNA methyltransferase 1-associated protein 1 | 4 | J:72247 |
| MGI:1918491 | Dmrtc2 | doublesex and mab-3 related transcription factor like family C2 | 7 | J:121537 / PMID:17447844 |
| MGI:99470 | Dnajc2 | DnaJ heat shock protein family (Hsp40) member C2 | 5 | J:60000 |
| MGI:1915326 | Dnajc9 | DnaJ heat shock protein family (Hsp40) member C9 | 14 | J:164563 |
| MGI:94912 | Dnmt1 | DNA methyltransferase 1 | 9 | J:72443 / PMID:11399088 / PMID:27841881 / PMID:16887828 |
| MGI:1261827 | Dnmt3a | DNA methyltransferase 3A | 12 | J:75938 / PMID:11934864 / PMID:17938196 / PMID:10555141 |
| MGI:1261819 | Dnmt3b | DNA methyltransferase 3B | 2 | J:75938 / PMID:11934864 / PMID:17938196 / PMID:10555141 |
| MGI:3649996 | Dnmt3c | DNA methyltransferase 3C | 2 | J:237199 / PMID:27856912 |
| MGI:1859287 | Dnmt3l | DNA methyltransferase 3-like | 10 | J:75938 / PMID:11934864 / PMID:16920095 / PMID:30834655 |
| MGI:2143886 | Dot1l | DOT1 like histone lysine methyltransferase | 10 | J:161428 |
| MGI:1352748 | Dpf1 | double PHD fingers 1 | 7 | J:320039 |
| MGI:109529 | Dpf2 | double PHD fingers 2 | 19 | J:320039 |
| MGI:1917377 | Dpf3 | double PHD fingers 3 | 12 | J:320039 |
| MGI:2157523 | Dppa2 | developmental pluripotency associated 2 | 16 | J:178324 / PMID:21896782 |
| MGI:1920958 | Dppa3 | developmental pluripotency-associated 3 | 6 | J:161428 |
| MGI:1913560 | Dpy30 | dpy-30, histone methyltransferase complex regulatory subunit | 17 | J:164563 |
| MGI:2656973 | Dtx3l | deltex 3-like, E3 ubiquitin ligase | 16 | J:164563 |
| MGI:1915440 | Dubr | Dppa2 upstream binding RNA | 16 | J:218442 / PMID:25686699 |
| MGI:1330299 | Dyrk1a | dual-specificity tyrosine phosphorylation regulated kinase 1a | 16 | J:346132 |
| MGI:95286 | Eed | embryonic ectoderm development | 7 | J:82790 / PMID:12627233 |
| MGI:1915293 | Eef1akmt1 | EEF1A alpha lysine methyltransferase 1 | 14 | J:346132 |
| MGI:1919346 | Eef1akmt2 | EEF1A lysine methyltransferase 2 | 7 | J:346132 |
| MGI:3645330 | Eef1akmt3 | EEF1A lysine methyltransferase 3 | 10 | J:346132 |
| MGI:1917761 | Eef2kmt | eukaryotic elongation factor 2 lysine methyltransferase | 16 | J:346132 |
| MGI:95295 | Egr1 | early growth response 1 | 18 | J:279331 |
| MGI:1924933 | Ehmt1 | euchromatic histone methyltransferase 1 | 2 | J:164563 |
| MGI:2148922 | Ehmt2 | euchromatic histone lysine Nmethyltransferase 2 | 17 | J:123364 / PMID:17212651 |
| MGI:105125 | Eif1 | eukaryotic translation initiation factor 1 | 11 | J:116443 / PMID:16789828 |
| MGI:1924203 | Emsy | EMSY, BRCA2-interacting transcriptional repressor | 7 | J:60000 |
| MGI:1919286 | Eny2 | ENY2 transcription and export complex 2 subunit | 15 | J:60000 |
| MGI:1201683 | Eomes | eomesodermin | 9 | J:266522 / PMID:30186101 |
| MGI:1276116 | Ep300 | E1A binding protein p300 | 15 | J:164563 |
| MGI:1276124 | Ep400 | E1A binding protein p400 | 5 | J:346132 |
| MGI:1278322 | Epc1 | enhancer of polycomb homolog 1 | 18 | J:66240 / PMID:10976108 |
| MGI:1278321 | Epc2 | enhancer of polycomb homolog 2 | 2 | J:60000 |
| MGI:2143991 | Epop | elongin BC and polycomb repressive complex 2 associated protein | 11 | J:238487 |
| MGI:1100494 | Ercc6 | excision repair cross-complementing rodent repair deficiency, complementation group 6 | 14 | J:320057 |
| MGI:2654144 | Ercc6l | excision repair cross-complementing rodent repair deficiency complementation group 6 like | X | J:346132 |
| MGI:1923501 | Ercc6l2 | excision repair cross-complementing rodent repair deficiency, complementation group 6 like 2 | 13 | J:346132 |
| MGI:1355322 | Exosc10 | exosome component 10 | 4 | J:116443 / PMID:16789828 |
| MGI:109344 | Eya1 | EYA transcriptional coactivator and phosphatase 1 | 1 | J:346132 |
| MGI:109341 | Eya2 | EYA transcriptional coactivator and phosphatase 2 | 2 | J:346132 |
| MGI:109339 | Eya3 | EYA transcriptional coactivator and phosphatase 3 | 4 | J:346132 |
| MGI:1337104 | Eya4 | EYA transcriptional coactivator and phosphatase 4 | 10 | J:60000 |
| MGI:1097695 | Ezh1 | enhancer of zeste 1 polycomb repressive complex 2 subunit | 11 | J:161428 |
| MGI:107940 | Ezh2 | enhancer of zeste 2 polycomb repressive complex 2 subunit | 6 | J:202816 / PMID:24105743 |
| MGI:2147968 | Ezhip | EZH inhibitory protein | X | J:281161 / PMID:31451685 |
| MGI:1918114 | Fam47c | family with sequence similarity 47 | X | J:161428 |
| MGI:2686227 | Fam47e | family with sequence similarity 47 | 5 | J:161428 |
| MGI:1351626 | Fam50a | family with sequence similarity 50 | X | J:161428 |
| MGI:1351640 | Fam50b | family with sequence similarity 50 | 13 | J:161428 |
| MGI:95486 | Fbl | fibrillarin | 7 | J:346132 |
| MGI:3034689 | Fbll1 | fibrillarin-like 1 | 11 | J:346132 |
| MGI:3039600 | Fbxl19 | F-box and leucine-rich repeat protein 19 | 7 | J:161428 |
| MGI:1918426 | Fbxo24 | F-box protein 24 | 5 | J:347878 / PMID:38470475 |
| MGI:1919115 | Fbxo30 | F-box protein 30 | 10 | J:290604 / PMID:30980108 |
| MGI:1916040 | Fendrr | Foxf1 adjacent non-coding developmental regulatory RNA | 8 | J:191565 / PMID:23369715 |
| MGI:2137612 | Fkbp6 | FK506 binding protein 6 | 5 | J:188133 |
| MGI:1925435 | Flicr | Foxp3 regulating long intergenic noncoding RNA | X | J:241346 / PMID:28396406 |
| MGI:1347472 | Foxa1 | forkhead box A1 | 12 | J:161021 |
| MGI:1347476 | Foxa2 | forkhead box A2 | 2 | J:60000 |
| MGI:1347477 | Foxa3 | forkhead box A3 | 7 | J:60000 |
| MGI:1891436 | Foxp3 | forkhead box P3 | X | J:117627 / PMID:17028180 |
| MGI:95583 | Fshr | follicle stimulating hormone receptor | 17 | J:61957 / PMID:10775161 / PMID:14680821 |
| MGI:1926128 | Ftx | Ftx transcript | X | J:168715 / PMID:21118898 / PMID:30237402 / PMID:21118898 |
| MGI:95663 | Gata3 | GATA binding protein 3 | 2 | J:176231 / PMID:21867929 / PMID:15087456 / PMID:15087456 |
| MGI:1914460 | Gatad1 | GATA zinc finger domain containing 1 | 5 | J:161428 |
| MGI:2384585 | Gatad2a | GATA zinc finger domain containing 2A | 8 | J:164563 |
| MGI:2443225 | Gatad2b | GATA zinc finger domain containing 2B | 3 | J:164563 |
| MGI:2141180 | Glmn | glomulin | 5 | J:164563 |
| MGI:1921272 | Glyr1 | glyoxylate reductase 1 homolog (Arabidopsis) | 16 | J:161428 |
| MGI:95777 | Gnas | GNAS complex locus | 2 | J:160293 / PMID:20427744 |
| MGI:1861674 | Gnasas1 | GNAS antisense RNA 1 | 2 | J:160293 / PMID:20427744 |
| MGI:104887 | Gpx1 | glutathione peroxidase 1 | 9 | J:164563 |
| MGI:104767 | Gpx4 | glutathione peroxidase 4 | 10 | J:69361 / PMID:11344099 |
| MGI:2141989 | Grwd1 | glutamate-rich WD repeat containing 1 | 7 | J:164563 |
| MGI:2152453 | Gsk3a | glycogen synthase kinase 3 alpha | 7 | J:173141 |
| MGI:1861437 | Gsk3b | glycogen synthase kinase 3 beta | 16 | J:173141 |
| MGI:2385191 | Gtf2b | general transcription factor IIB | 3 | J:346132 |
| MGI:2138937 | Gtf3c4 | general transcription factor IIIC, polypeptide 4 | 2 | J:346132 |
| MGI:95891 | H19 | H19 | 7 | J:42931 / PMID:9294195 |
| MGI:95893 | H1f0 | H1.0 linker histone | 15 | J:164563 |
| MGI:1931523 | H1f1 | H1.1 linker histone, cluster member | 13 | J:72247 |
| MGI:1931526 | H1f2 | H1.2 linker histone | 13 | J:83954 / PMID:12808097 |
| MGI:107502 | H1f3 | H1.3 linker histone | 13 | J:225113 / PMID:22701719 / PMID:12808097 |
| MGI:1931527 | H1f4 | H1.4 linker histone | 13 | J:83954 [PMID:12808097] |
| MGI:1861461 | H1f5 | H1.5 linker histone | 13 | J:164563 |
| MGI:1888530 | H1f6 | H1.6 linker histone, cluster member | 13 | J:72247 |
| MGI:1917319 | H1f7 | H1.7 linker histone | 15 | J:96828 |
| MGI:2176207 | H1f8 | H1.8 linker histone | 6 | J:74931 / PMID:11171391 |
| MGI:2136691 | H1f9 | H1.9 linker histone | 11 | J:164563 |
| MGI:3642445 | H2ab1 | H2A.B variant histone 1 | X | J:161428 |
| MGI:3644980 | H2ab2 | H2A.B variant histone 2 | X | J:161428 |
| MGI:3644875 | H2ab3 | H2A.B variant histone 3 | X | J:161428 |
| MGI:2448285 | H2ac1 | H2A clustered histone 1 | 13 | J:210169 / PMID:24506885 |
| MGI:2448309 | H2ac10 | H2A clustered histone 10 | 13 | J:161428 |
| MGI:2448293 | H2ac11 | H2A clustered histone 11 | 13 | J:161428 |
| MGI:2448295 | H2ac12 | H2A clustered histone 12 | 13 | J:161428 |
| MGI:2448457 | H2ac13 | H2A clustered histone 13 | 13 | J:161428 |
| MGI:2448297 | H2ac15 | H2A clustered histone 15 | 13 | J:161428 |
| MGI:2448283 | H2ac19 | H2A clustered histone 19 | 3 | J:161428 |
| MGI:2448316 | H2ac20 | H2A clustered histone 20 | 3 | J:161428 |
| MGI:2448314 | H2ac21 | H2A clustered histone 21 | 3 | J:161428 |
| MGI:2448300 | H2ac22 | H2A clustered histone 22 | 13 | J:161428 |
| MGI:2448302 | H2ac23 | H2A clustered histone 23 | 13 | J:161428 |
| MGI:3710573 | H2ac24 | H2A clustered histone 24 | 13 | J:161428 |
| MGI:2448458 | H2ac25 | H2A clustered histone 25 | 11 | J:164563 |
| MGI:2448306 | H2ac4 | H2A clustered histone 4 | 13 | J:161428 |
| MGI:2448287 | H2ac6 | H2A clustered histone 6 | 13 | J:161428 |
| MGI:2448289 | H2ac7 | H2A clustered histone 7 | 13 | J:161428 |
| MGI:2448290 | H2ac8 | H2A clustered histone 8 | 13 | J:161428 |
| MGI:3606192 | H2aj | H2J.A histone | 6 | J:161428 |
| MGI:3650131 | H2al1b | H2A histone family member L1B | X | J:161428 |
| MGI:3649617 | H2al1e | H2A histone family member L1E | X | J:161428 |
| MGI:3649874 | H2al1f | H2A histone family member L1F | X | J:161428 |
| MGI:3643273 | H2al1j | H2A histone family member L1J | X | J:161428 |
| MGI:3710586 | H2al1k | H2A histone family member L1K | X | J:161428 |
| MGI:1923633 | H2al1m | H2A histone family member L1M | X | J:161428 |
| MGI:3643774 | H2al1n | H2A histone family member L1N | X | J:161428 |
| MGI:3643069 | H2al1o | H2A histone family member L1O | X | J:161428 |
| MGI:1915481 | H2al2a | H2A histone family member L2A | 2 | J:119495 / PMID:17261847 |
| MGI:3710623 | H2al2b | H2A histone family member L2B | Y | J:161428 |
| MGI:1922521 | H2al3 | H2A histone family member L3 | X | J:161428 |
| MGI:1914584 | H2ap | H2A.P histone | X | J:161428 |
| MGI:102688 | H2ax | H2A.X variant histone | 9 | J:161428 |
| MGI:1888388 | H2az1 | H2A.Z variant histone 1 | 3 | J:320332 |
| MGI:1924855 | H2az2 | H2A.Z histone variant 2 | 11 | J:320332 |
| MGI:2448375 | H2bc1 | H2B clustered histone 1 | 13 | J:210169 / PMID:24506885 / PMID:17261847 |
| MGI:1097686 | H3f3a | H3.3 histone A | 1 | J:225909 / PMID:26159997 |
| MGI:1101782 | H3f3a-ps1 | H3.3 histone A | X | J:224673 / PMID:26388943 |
| MGI:1101758 | H3f3a-ps2 | H3.3 histone A | 16 | J:224673 / PMID:26388943 |
| MGI:1101768 | H3f3b | H3.3 histone B | 11 | J:225909 / PMID:26159997 |
| MGI:3650546 | H3f3c | H3 histone | 2 | J:224673 / PMID:26388943 |
| MGI:3651326 | H3f4 | H3.4 histone | 11 | J:224673 / PMID:26388943 |
| MGI:3651714 | H3f5 | H3.5 histone | 4 | J:224673 / PMID:26388943 |
| MGI:2140113 | H4c14 | H4 clustered histone 14 | 3 | J:161428 |
| MGI:1194498 | Haspin | histone H3 associated protein kinase | 11 | J:346132 |
| MGI:96013 | Hat1 | histone aminotransferase 1 | 2 | J:72247 |
| MGI:105942 | Hcfc1 | host cell factor C1 | X | J:161428 |
| MGI:1915183 | Hcfc2 | host cell factor C2 | 10 | J:161428 |
| MGI:108086 | Hdac1 | histone deacetylase 1 | 4 | J:164563 |
| MGI:2158340 | Hdac10 | histone deacetylase 10 | 15 | J:346132 |
| MGI:2385252 | Hdac11 | histone deacetylase 11 | 6 | J:161428 |
| MGI:1097691 | Hdac2 | histone deacetylase 2 | 10 | J:164563 |
| MGI:1343091 | Hdac3 | histone deacetylase 3 | 18 | J:164563 |
| MGI:3036234 | Hdac4 | histone deacetylase 4 | 1 | J:164563 |
| MGI:1333784 | Hdac5 | histone deacetylase 5 | 11 | J:161428 |
| MGI:1333752 | Hdac6 | histone deacetylase 6 | X | J:164563 |
| MGI:1891835 | Hdac7 | histone deacetylase 7 | 15 | J:161428 |
| MGI:1917565 | Hdac8 | histone deacetylase 8 | X | J:161428 |
| MGI:1931221 | Hdac9 | histone deacetylase 9 | 12 | J:164563 |
| MGI:1194494 | Hdgf | heparin binding growth factor | 3 | J:161428 |
| MGI:1194493 | Hdgfl1 | HDGF like 1 | 13 | J:161428 |
| MGI:1194492 | Hdgfl2 | HDGF like 2 | 17 | J:161428 |
| MGI:1352760 | Hdgfl3 | HDGF like 3 | 7 | J:161428 |
| MGI:106209 | Hells | helicase | 19 | J:72618 / PMID:11711429 / PMID:15448183 |
| MGI:2685008 | Hipk4 | homeodomain interacting protein kinase 4 | 7 | J:346132 |
| MGI:99430 | Hira | histone cell cycle regulator | 16 | J:161428 |
| MGI:2685821 | Hjurp | Holliday junction recognition protein | 1 | J:161428 |
| MGI:1196437 | Hltf | helicase-like transcription factor | 3 | J:346132 |
| MGI:1914117 | Hmg20a | high mobility group 20A | 9 | J:60000 |
| MGI:1341190 | Hmg20b | high mobility group 20B | 10 | J:69281 |
| MGI:96160 | Hmga1 | high mobility group AT-hook 1 | 17 | J:190069 / PMID:22797695 |
| MGI:101761 | Hmga2 | high mobility group AT-hook 2 | 10 | J:164563 |
| MGI:96113 | Hmgb1 | high mobility group box 1 | 5 | J:161428 |
| MGI:96157 | Hmgb2 | high mobility group box 2 | 8 | J:161428 / PMID: 37573836 / PMID: 27226577 |
| MGI:1098219 | Hmgb3 | high mobility group box 3 | X | PMID: 12714519 |
| MGI:1916567 | Hmgb4 | high-mobility group box 4 | 4 | J:161428 / PMID: 33069132 |
| MGI:96120 | Hmgn1 | high mobility group nucleosomal binding domain 1 | 16 | J:100282 / PMID:12660172 |
| MGI:96136 | Hmgn2 | high mobility group nucleosomal binding domain 2 | 4 | J:161428 |
| MGI:2138069 | Hmgn3 | high mobility group nucleosomal binding domain 3 | 9 | J:161428 |
| MGI:1355295 | Hmgn5 | high-mobility group nucleosome binding domain 5 | X | J:212215 / PMID:24392144 |
| MGI:98504 | Hnf1a | HNF1 homeobox A | 5 | J:118400 / PMID:11733582 |
| MGI:1858195 | Hnrnpu | heterogeneous nuclear ribonucleoprotein U | 1 | J:164563 |
| MGI:3826586 | Hotair | HOX transcript antisense RNA (non-protein coding) | 15 | J:203783 / PMID:24075995 |
| MGI:109369 | Hp1bp3 | heterochromatin protein 1 | 4 | J:161428 |
| MGI:1919862 | Hpf1 | histone PARylation factor 1 | 8 | J:164563 |
| MGI:96223 | Hr | lysine demethylase and nuclear receptor corepressor | 14 | J:346132 |
| MGI:1919709 | Htatsf1 | HIV TAT specific factor 1 | X | J:346132 |
| MGI:1926884 | Huwe1 | HECT, UBA and WWE domain containing 1 | X | J:346132 |
| MGI:2651552 | Hymai | hydatidiform mole associated and imprinted transcript | 10 | J:187821 / PMID:22723905 |
| MGI:1913441 | Ier3ip1 | immediate early response 3 interacting protein 1 | 18 | J:336782 / PMID:36322741 |
| MGI:96428 | Ifi203 | interferon activated gene 203 | 1 | J:164563 |
| MGI:3840117 | Ifi203-ps | interferon activated gene 203, pseudogene | 1 | J:164563 |
| MGI:3646410 | Ifi206 | interferon activated gene 206 | 1 | J:164563 |
| MGI:2138302 | Ifi207 | interferon activated gene 207 | 1 | J:164563 |
| MGI:2442822 | Ifi208 | interferon activated gene 208 | 1 | J:164563 |
| MGI:2138243 | Ifi209 | interferon activated gene 209 | 1 | J:164563 |
| MGI:3695276 | Ifi213 | interferon activated gene 213 | 1 | J:164563 |
| MGI:3584522 | Ifi214 | interferon activated gene 214 | 1 | J:164563 |
| MGI:1342540 | Ikzf1 | IKAROS family zinc finger 1 | 11 | J:60000 |
| MGI:1916510 | Ing2 | inhibitor of growth family, member 2 | 8 | J:167311 |
| MGI:1919027 | Ing3 | inhibitor of growth family, member 3 | 6 | J:346132 |
| MGI:107307 | Ing4 | inhibitor of growth family | 6 | J:164563 |
| MGI:1922816 | Ing5 | inhibitor of growth family | 1 | J:164563 |
| MGI:1915392 | Ino80 | INO80 complex subunit | 2 | J:161428 |
| MGI:1917270 | Ino80b | INO80 complex subunit B | 6 | J:164563 |
| MGI:2443014 | Ino80c | INO80 complex subunit C | 18 | J:161428 |
| MGI:3027003 | Ino80d | INO80 complex subunit D | 1 | J:164563 |
| MGI:2141881 | Ino80e | INO80 complex subunit E | 7 | J:164563 |
| MGI:1096873 | Irf4 | interferon regulatory factor 4 | 13 | J:105503 / PMID:16428437 |
| MGI:1914983 | Itgb3bp | integrin beta 3 binding protein (beta3endonexin) | 4 | J:72247 |
| MGI:1920723 | Iws1 | IWS1 | 18 | J:164563 |
| MGI:1925835 | Jade1 | jade family PHD finger 1 | 3 | J:346132 |
| MGI:1924151 | Jade2 | jade family PHD finger 2 | 11 | J:346132 |
| MGI:96629 | Jak2 | Janus kinase 2 | 19 | J:346132 |
| MGI:1922855 kdm5b | Jarid1b | lysine demethylase 5B | 1 | PMID: 22615382 |
| MGI:99781 | Jarid1c | lysine demethylase 5C | X | PMID: 18155621 |
| MGI:99780 | Jarid1d | lysine demethylase 5D | Y | PMID: 17320162 |
| MGI:104813 | Jarid2 | jumonji and AT-rich interaction domain containing 2 | 13 | J:240642 / PMID:27892467 |
| MGI:1932093 | Jdp2 | Jun dimerization protein 2 | 12 | J:139241 / PMID:17464331 |
| MGI:98847 | Jmjd1a | jumonji domain containing 1A | 6 | PMID: 24214985 |
| MGI:1923356 | Jmjd1b | jumonji domain containing 1B | 18 | PMID: 32070414 |
| MGI:1918614 | Jmjd1c | jumonji domain containing 1C | 10 | J:346132 |
| MGI:2446210 | Jmjd2a | lysine (K)-specific demethylase 4A | 4 | PMID: 35000672 |
| MGI:2442355 | Jmjd2b | lysine (K)-specific demethylase 4B | 17 | PMID: 22133676 |
| MGI:1924054 | Jmjd2c | lysine (K)-specific demethylase 4C | 4 | PMID: 17277772 |
| MGI:3606484 | Jmjd2d | lysine (K)-specific demethylase 4D | 9 | PMID: 17207460 |
| MGI:2448492 | Jmjd3 | KDM1 lysine (K)-specific demethylase 6B | 11 | PMID: 35672304 |
| MGI:1924285 | Jmjd5 | lysine (K)-specific demethylase 8 | 7 | PMID: 22402282 |
| MGI:1858910 | Jmjd6 | jumonji domain containing 6 | 11 | J:164563 |
| MGI:2180008 | Jpx | Jpx transcript | X | J:212497 / PMID:24613346 / PMID:21029862 |
| MGI:1923969 | Kansl1 | KAT8 regulatory NSL complex subunit 1 | 11 | J:60000 |
| MGI:1916862 | Kansl2 | KAT8 regulatory NSL complex subunit 2 | 15 | J:60000 |
| MGI:1918055 | Kansl3 | KAT8 regulatory NSL complex subunit 3 | 1 | J:60000 |
| MGI:1917264 | Kat14 | lysine acetyltransferase 14 | 2 | J:346132 |
| MGI:1343101 | Kat2a | K(lysine) acetyltransferase 2A | 11 | J:161428 |
| MGI:1343094 | Kat2b | K(lysine) acetyltransferase 2B | 17 | J:161428 |
| MGI:1932051 | Kat5 | K(lysine) acetyltransferase 5 | 19 | J:164563 |
| MGI:2442415 | Kat6a | K(lysine) acetyltransferase 6A | 8 | J:72247 |
| MGI:1858746 | Kat6b | K(lysine) acetyltransferase 6B | 14 | J:72247 |
| MGI:2182799 | Kat7 | K(lysine) acetyltransferase 7 | 11 | J:164563 |
| MGI:1915023 | Kat8 | K(lysine) acetyltransferase 8 | 7 | J:164563 |
| MGI:1926855 | Kcnq1ot1 | KCNQ1 overlapping transcript 1 | 7 | J:92776 / PMID:15340049 / PMID:20573698 / PMID:15340049 |
| MGI:1196256 | Kdm1a | lysine (K)-specific demethylase 1A | 4 | J:208295 [PMID:24217620] |
| MGI:2145261 | Kdm1b | lysine (K)-specific demethylase 1B | 13 | J:152382 [PMID:19727073] |
| MGI:1354736 | Kdm2a | lysine (K)-specific demethylase 2A | 19 | J:161428 |
| MGI:1354737 | Kdm2b | lysine (K)-specific demethylase 2B | 5 | J:161428 |
| MGI:98847 | Kdm3a | lysine (K)-specific demethylase 3A | 6 | J:346132 |
| MGI:1923356 | Kdm3b | KDM3B lysine (K)-specific demethylase 3B | 18 | J:346132 |
| MGI:2446210 | Kdm4a | lysine (K)-specific demethylase 4A | 4 | J:161428 |
| MGI:2442355 | Kdm4b | lysine (K)-specific demethylase 4B | 17 | J:161428 |
| MGI:1924054 | Kdm4c | lysine (K)-specific demethylase 4C | 4 | J:161428 |
| MGI:3606484 | Kdm4d | lysine (K)-specific demethylase 4D | 9 | J:161428 |
| MGI:2136980 | Kdm5a | lysine demethylase 5A | 6 | J:161428 |
| MGI:1922855 | Kdm5b | lysine demethylase 5B | 1 | J:161428 |
| MGI:99781 | Kdm5c | lysine demethylase 5C | X | J:161428 |
| MGI:99780 | Kdm5d | lysine demethylase 5D | Y | J:161428 |
| MGI:1095419 | Kdm6a | lysine (K)-specific demethylase 6A | X | J:166998 |
| MGI:2448492 | Kdm6b | KDM1 lysine (K)-specific demethylase 6B | 11 | J:166998 |
| MGI:2443388 | Kdm7a | lysine (K)-specific demethylase 7A | 6 | J:161428 |
| MGI:1924285 | Kdm8 | lysine (K)-specific demethylase 8 | 7 | J:346132 |
| MGI:1342771 | Klf1 | Kruppel-like transcription factor 1 (erythroid) | 8 | J:93146 / PMID:15489291 |
| MGI:1342772 | Klf2 | Kruppel-like transcription factor 2 (lung) | 8 | J:185718 / PMID:22482507 |
| MGI:96995 | Kmt2a | lysine (K)-specific methyltransferase 2A | 9 | J:109102 / PMID:16618927 |
| MGI:109565 | Kmt2b | lysine (K)-specific methyltransferase 2B | 7 | J:166778 / PMID:20808952 |
| MGI:2444959 | Kmt2c | lysine (K)-specific methyltransferase 2C | 5 | J:346132 |
| MGI:2682319 | Kmt2d | lysine (K)-specific methyltransferase 2D | 15 | J:166778 |
| MGI:1924825 | Kmt2e | lysine (K)-specific methyltransferase 2E | 5 | J:145445 |
| MGI:1915206 | Kmt5a | lysine methyltransferase 5A | 5 | J:346132 |
| MGI:2444557 | Kmt5b | lysine methyltransferase 5B | 19 | J:346132 |
| MGI:2385262 | Kmt5c | lysine methyltransferase 5C | 7 | J:346132 |
| MGI:2141165 | Kpna7 | karyopherin subunit alpha 7 | 5 | J:244973 / PMID:20699224 |
| MGI:2676663 | L3mbtl1 | L3MBTL1 histone methyl-lysine binding protein | 2 | J:164563 |
| MGI:2443584 | L3mbtl2 | L3MBTL2 polycomb repressive complex 1 subunit | 15 | J:227373 / PMID:22770845 |
| MGI:2143628 | L3mbtl3 | L3MBTL3 histone methyl-lysine binding protein | 10 | J:60000 |
| MGI:2444889 | L3mbtl4 | L3MBTL4 histone methyl-lysine binding protein | 17 | J:60000 |
| MGI:99914 | Lamc1 | laminin | 1 | J:239434 / PMID:27234308 |
| MGI:2443930 | Lcor | ligand dependent nuclear receptor corepressor | 19 | J:262237 |
| MGI:1915864 | Letmd1 | LETM1 domain containing 1 | 15 | J:321533 / PMID:34910916 |
| MGI:96785 | Lhx2 | LIM homeobox protein 2 | 2 | J:237908 |
| MGI:2140902 | Lin54 | lin-54 DREAM MuvB core complex component | 5 | J:164563 |
| MGI:96794 | Lmna | lamin A | 3 | J:161428 |
| MGI:96795 | Lmnb1 | lamin B1 | 18 | J:161428 |
| MGI:96796 | Lmnb2 | lamin B2 | 10 | J:161428 |
| MGI:2137913 | Loxl2 | lysyl oxidase-like 2 | 14 | J:164563 |
| MGI:2445214 | Lrif1 | ligand dependent nuclear receptor interacting factor 1 | 3 | J:164563 |
| MGI:1918985 | Lrwd1 | leucine-rich repeats and WD repeat domain containing 1 | 5 | J:161428 |
| MGI:1919540 | Lsm11 | U7 snRNP-specific Sm-like protein LSM11 | 11 | J:164563 |
| MGI:1315200 | M1ap | meiosis 1 associated protein | 6 | J:52798 |
| MGI:1349392 | Macroh2a1 | macroH2A.1 histone | 13 | J:89037 / PMID:11331621 |
| MGI:3037658 | Macroh2a2 | macroH2A.2 histone | 10 | J:89037 / PMID:11331621 |
| MGI:2138453 | Mael | maelstrom spermatogenic transposon silencer | 1 | J:155865 |
| MGI:1346877 | Map3k7 | mitogen-activated protein kinase kinase kinase 7 | 4 | J:346132 |
| MGI:1921799 | Mau2 | MAU2 sister chromatid cohesion factor | 8 | PMID: 32433956 |
| MGI:1333811 | Mbd1 | methyl-CpG binding domain protein 1 | 18 | J:161428 |
| MGI:1333813 | Mbd2 | methyl-CpG binding domain protein 2 | 18 | J:164563 |
| MGI:1333812 | Mbd3 | methyl-CpG binding domain protein 3 | 10 | J:342607 |
| MGI:1920753 | Mbd3l1 | methyl-CpG binding domain protein 3-like 1 | 9 | J:161428 |
| MGI:2158460 | Mbd3l2 | methyl-CpG binding domain protein 3-like 2 | 9 | J:161428 |
| MGI:2143977 | Mbtd1 | mbt domain containing 1 | 11 | J:60000 |
| MGI:105380 | Mcm2 | minichromosome maintenance complex component 2 | 6 | J:72594 / PMID:11568184 |
| MGI:1930089 | Mcm3ap | minichromosome maintenance complex component 3 associated protein | 10 | J:164563 |
| MGI:2384752 | Mcrip1 | MAPK regulated corepressor interacting protein 1 | 11 | J:278092 / PMID:31240265 |
| MGI:1858420 | Mcrs1 | microspherule protein 1 | 15 | J:164563 |
| MGI:3525201 | Mdc1 | mediator of DNA damage checkpoint 1 | 17 | J:346132 |
| MGI:1917338 | Meaf6 | MYST/Esa1-associated factor 6 | 4 | J:346132 |
| MGI:95457 | Mecom | MDS1 and EVI1 complex locus | 3 | J:269562 |
| MGI:99918 | Mecp2 | methyl CpG binding protein 2 | X | J:98000 / PMID:15757975 / PMID:17532643 / PMID:15608638 |
| MGI:1344385 | Med24 | mediator complex subunit 24 | 11 | J:346132 |
| MGI:1202886 | Meg3 | maternally expressed 3 | 12 | J:290525 / PMID:26005002 / PMID:20610486 / PMID:23195472 |
| MGI:1316736 | Men1 | multiple endocrine neoplasia 1 | 19 | J:105708 / PMID:16415155 / PMID:15044367 |
| MGI:1914349 | Mettl21a | methyltransferase 21A, HSPA lysine | 1 | J:346132 |
| MGI:2384301 | Mettl22 | methyltransferase 22, Kin17 lysine | 16 | J:346132 |
| MGI:1921569 | Mettl23 | methyltransferase like 23 | 11 | J:254851 |
| MGI:1927165 | Mettl3 | methyltransferase 3, N6-adenosinemethyltransferase complex catalytic subunit | 14 | J:164563 |
| MGI:1924031 | Mettl4 | methyltransferase 4, N6-adenosine | 17 | J:278437 |
| MGI:2385142 | Mettl8 | methyltransferase 8, methylcytidine | 2 | J:346132 |
| MGI:2140270 | Mexis | macrophage expressed LXRa(NR1H3)dependent amplifier of Abca1 transcription lncRNA | 4 | J:264834 / PMID:29431742 |
| MGI:3642848 | Mhrt | myosin heavy chain associated RNA transcript | 14 | J:213053 / PMID:25119045 |
| MGI:1918398 | Mier1 | MEIR1 treanscription regulator | 4 | J:164563 |
| MGI:1917677 | Mier2 | MIER family member 2 | 10 | J:346132 |
| MGI:2676812 | Mir127 | microRNA 127 | 12 | J:144511 / PMID:19126398 / PMID:26138477 |
| MGI:2676821 | Mir136 | microRNA 136 | 12 | J:144511 / PMID:19126398 |
| MGI:3718461 | Mir208b | microRNA 208b | 14 | J:211907 / PMID:24137001 |
| MGI:3691603 | Mir744 | microRNA 744 | 11 | J:197739 / PMID:22053081 |
| MGI:3781106 | Mirg | miRNA containing gene | 12 | J:290525 / PMID:26005002 |
| MGI:1913828 | Mis18a | MIS18 kinetochore protein A | 16 | J:188023 / PMID:22516971 |
| MGI:106035 | Mki67 | antigen identified by monoclonal antibody Ki 67 | 7 | J:235309 |
| MGI:1917372 | Mllt3 | myeloid/lymphoid or mixed-lineage leukemia; translocated to, 3 | 4 | J:164563 |
| MGI:1935145 | Mllt6 | myeloid/lymphoid or mixed-lineage leukemia; translocated to | 11 | J:222358 / PMID:21546577 |
| MGI:2684980 | Mms22l | MMS22-like, DNA repair protein | 4 | J:60000 |
| MGI:3780953 | Mndal | myeloid nuclear differentiation antigen like | 1 | J:164563 |
| MGI:1316740 | Morc1 | microrchidia 1 | 16 | J:225677 / PMID:25503965 |
| MGI:1921772 | Morc2a | microrchidia 2A | 11 | J:164563 |
| MGI:3045293 | Morc2b | microrchidia 2B | 17 | J:164563 |
| MGI:1096551 | Morf4l1 | mortality factor 4 like 1 | 9 | J:68893 / PMID:11290425 |
| MGI:1927167 | Morf4l2 | mortality factor 4 like 2 | X | J:60000 |
| MGI:97052 | Mos | Moloney sarcoma oncogene | 4 | J:32974 |
| MGI:97054 | Mov10 | Mov10 RISC complex RNA helicase | 3 | J:164563 |
| MGI:1891384 | Mov10l1 | Mov10 like RISC complex RNA helicase 1 | 15 | J:161125 |
| MGI:1922589 | Mphosph8 | M-phase phosphoprotein 8 | 14 | J:164563 |
| MGI:1100512 | Mre11a | MRE11A homolog A, double strand break repair nuclease | 9 | J:164563 |
| MGI:1920497 | Mrgbp | MRG/MORF4L binding protein | 2 | J:60000 |
| MGI:1921276 | Msl1 | male specific lethal 1 | 11 | J:277869 |
| MGI:1925103 | Msl2 | MSL complex subunit 2 | 9 | J:344688 |
| MGI:1341851 | Msl3 | MSL complex subunit 3 | X | J:346132 |
| MGI:96080 | Mst1 | macrophage stimulating 1 (hepatocyte growth factor-like) | 9 | J:346132 |
| MGI:2150037 | Mta1 | metastasis associated 1 | 12 | J:342607 |
| MGI:1346340 | Mta2 | metastasis-associated gene family | 19 | J:87046 / PMID:14645126 / PMID:20720167 |
| MGI:2151172 | Mta3 | metastasis associated 3 | 17 | J:164563 |
| MGI:105050 | Mtf2 | metal response element binding transcription factor 2 | 5 | J:161428 |
| MGI:106639 | Mthfr | methylenetetrahydrofolate reductase | 4 | J:164563 |
| MGI:106181 | Mybbp1a | MYB binding protein (P160) 1a | 11 | J:320057 |
| MGI:99925 | Mybl1 | myeloblastosis oncogene-like 1 | 1 | J:198228 |
| MGI:97250 | Myc | myelocytomatosis oncogene | 15 | J:164563 / PMID: 16724113 |
| MGI:97357 | Mycn | v-myc avian myelocytomatosis viral related oncogene, neuroblastoma derived | 12 | J:173141 |
| MGI:108005 | Myd88 | myeloid differentiation primary response gene 88 | 9 | J:136566 / PMID:18319258 |
| MGI:106612 | Myo1c | myosin IC | 11 | J:320057 |
| MGI:2137495 | Myocd | myocardin | 11 | J:95385 / PMID:15601857 |
| MGI:2444584 | Mysm1 | myb-like | 4 | J:161428 |
| MGI:1915018 | N6amt1 | N-6 adenine-specific DNA methyltransferase 1 (putative) | 16 | J:164563 |
| MGI:1918249 | Naa40 | N(alpha)-acetyltransferase 40, NatD catalytic subunit | 19 | J:346132 |
| MGI:1919367 | Naa50 | N(alpha)-acetyltransferase 50, NatE catalytic subunit | 16 | J:346132 |
| MGI:1922013 | Naa60 | N(alpha)-acetyltransferase 60 | 16 | J:164563 |
| MGI:1855693 | Nap1l1 | nucleosome assembly protein 1-like 1 | 10 | J:161428 |
| MGI:106654 | Nap1l2 | nucleosome assembly protein 1-like 2 | X | J:161428 |
| MGI:1859565 | Nap1l3 | nucleosome assembly protein 1-like 3 | X | J:161428 |
| MGI:1316687 | Nap1l4 | nucleosome assembly protein 1-like 4 | 7 | J:161428 |
| MGI:1923555 | Nap1l5 | nucleosome assembly protein 1-like 5 | 6 | J:72247 |
| MGI:1355328 | Nasp | nuclear autoantigenic sperm protein (histonebinding) | 4 | J:161428 |
| MGI:2136449 | Nat8f3 | N-acetyltransferase 8 (GCN5-related) family member 3 | 6 | J:346132 |
| MGI:3782661 | Nat8f7 | N-acetyltransferase 8 (GCN5-related) family member 7 | 6 | J:346132 |
| MGI:1351625 | Nbn | nibrin | 4 | J:164563 |
| MGI:97286 | Ncl | nucleolin | 1 | PMID: 17157503 |
| MGI:1276523 | Ncoa1 | nuclear receptor coactivator 1 | 12 | J:346132 |
| MGI:1276535 | Ncoa3 | nuclear receptor coactivator 3 | 2 | J:346132 |
| MGI:1929915 | Ncoa6 | nuclear receptor coactivator 6 | 2 | J:346132 |
| MGI:1349717 | Ncor1 | nuclear receptor co-repressor 1 | 11 | J:60000 |
| MGI:97290 | Ndn | necdin | 7 | J:57890 / PMID:10508517 |
| MGI:1859333 | Nfat5 | nuclear factor of activated T cells 5 | 8 | J:164563 |
| MGI:1931595 | Nfkbiz | nuclear factor of kappa light polypeptide gene enhancer in B cells inhibitor | 16 | J:136566 / PMID:18319258 / PMID:25124037 / PMID:27129283 |
| MGI:2442410 | Nfrkb | nuclear factor related to kappa B binding protein | 9 | J:164563 |
| MGI:1913976 | Nipbl | NIPBL cohesin loading factor | 15 | J:164563 |
| MGI:106184 | Npm1 | nucleophosmin 1 | 11 | J:161428 |
| MGI:1890811 | Npm2 | nucleophosmin/nucleoplasmin 2 | 14 | J:161428 |
| MGI:894653 | Npm3 | nucleoplasmin 3 | 19 | J:161428 |
| MGI:95824 | Nr3c1 | nuclear receptor subfamily 3, group C, member 1 | 18 | J:155856 |
| MGI:2670969 | Nrde2 | nrde-2 necessary for RNA interference, domain containing | 12 | J:161428 |
| MGI:1276545 | Nsd1 | nuclear receptor-binding SET-domain protein 1 | 13 | J:346132 |
| MGI:1276574 | Nsd2 | nuclear receptor binding SET domain protein 2 | 5 | J:346132 |
| MGI:2142581 | Nsd3 | nuclear receptor binding SET domain protein 3 | 8 | J:346132 |
| MGI:1913867 | Ntmt1 | N-terminal Xaa-Pro-Lys N-methyltransferase 1 | 2 | J:346132 |
| MGI:1934811 | Nucks1 | nuclear casein kinase and cyclin-dependent kinase substrate 1 | 1 | J:211777 |
| MGI:1858232 | Nudt5 | nudix hydrolase 5 | 2 | J:164563 |
| MGI:1932139 | Oga | O-GlcNAcase | 19 | J:346132 |
| MGI:1097693 | Ogg1 | 8-oxoguanine DNA-glycosylase 1 | 6 | J:342605 |
| MGI:1339639 | Ogt | O-linked N-acetylglucosamine (GlcNAc) transferase (UDP-N-acetylglucosamine:polypeptide-Nacetylglucosaminyl transferase) | X | J:60000 |
| MGI:1917895 | Oip5 | Opa interacting protein 5 | 2 | J:161428 |
| MGI:1922908 | Pabpc1l | poly(A) binding protein | 2 | J:224534 / PMID:26134869 |
| MGI:1338892 | Padi2 | peptidyl arginine deiminase | 4 | J:161428 |
| MGI:1338898 | Padi4 | peptidyl arginine deiminase | 4 | J:161428 |
| MGI:1339975 | Pak1 | p21 (RAC1) activated kinase 1 | 7 | J:164563 |
| MGI:3712326 | Parp10 | poly (ADP-ribose) polymerase family, member 10 | 15 | J:342605 |
| MGI:1341112 | Parp2 | poly (ADP-ribose) polymerase family, member 2 | 14 | J:164563 |
| MGI:97490 | Pax6 | paired box 6 | 2 | J:266522 / PMID:30186101 |
| MGI:97491 | Pax7 | paired box 7 | 4 | J:188254 / PMID:23070814 / PMID:22862948 |
| MGI:1890430 | Paxip1 | PAX interacting (with transcription-activation domain) protein 1 | 5 | J:342605 |
| MGI:1923998 | Pbrm1 | polybromo 1 | 14 | J:161428 |
| MGI:108202 | Pcbp2 | poly(rC) binding protein 2 | 15 | J:164563 |
| MGI:1917087 | Pcgf1 | polycomb group ring finger 1 | 6 | J:164563 |
| MGI:99161 | Pcgf2 | polycomb group ring finger 2 | 11 | J:109525 / PMID:16687444 |
| MGI:1916837 | Pcgf3 | polycomb group ring finger 3 | 5 | J:243420 |
| MGI:1923505 | Pcgf5 | polycomb group ring finger 5 | 19 | J:243420 |
| MGI:1918291 | Pcgf6 | polycomb group ring finger 6 | 19 | J:164563 |
| MGI:2443003 | Pcid2 | PCI domain containing 2 | 8 | J:256297 |
| MGI: 2140945 | Pds5 | PDS5 cohesin associated factor | 5 | PMID: 36129789 |
| MGI:1098283 | Per1 | period circadian clock 1 | 11 | J:173532 |
| MGI:1195265 | Per2 | period circadian clock 2 | 1 | J:173532 |
| MGI:97572 | Phb1 | prohibitin 1 | 11 | J:342605 |
| MGI:103248 | Phc1 | polyhomeotic 1 | 6 | J:164563 |
| MGI:98647 | Phf1 | PHD finger protein 1 | 17 | J:164563 |
| MGI:1919307 | Phf10 | PHD finger protein 10 | 17 | J:320039 |
| MGI:2446217 | Phf13 | PHD finger protein 13 | 4 | J:60000 |
| MGI:1923539 | Phf14 | PHD finger protein 14 | 6 | J:346132 |
| MGI:1921266 | Phf19 | PHD finger protein 19 | 2 | J:183475 |
| MGI:1338034 | Phf2 | PHD finger protein 2 | 13 | J:164563 |
| MGI:2444148 | Phf20 | PHD finger protein 20 | 2 | J:60000 |
| MGI:2384756 | Phf21a | PHD finger protein 21A | 2 | J:60000 |
| MGI:2444341 | Phf8 | PHD finger protein 8 | X | J:346132 |
| MGI:1916095 | Pih1d1 | PIH1 domain containing 1 | 7 | J:342605 |
| MGI:1206581 | Pik3ca | phosphatidylinositol-4,5-bisphosphate 3kinase catalytic subunit alpha | 3 | J:173141 |
| MGI:1928897 | Piwil1 | piwi-like RNA-mediated gene silencing 1 | 5 | J:182800 |
| MGI:1930036 | Piwil2 | piwi-like RNA-mediated gene silencing 2 | 14 | J:199259 |
| MGI:3041167 | Piwil4 | piwi-like RNA-mediated gene silencing 4 | 9 | J:225677 / PMID:25503965 |
| MGI:97591 | Pkm | pyruvate kinase, muscle | 9 | J:346132 |
| MGI:108022 | Pkn1 | protein kinase N1 | 8 | J:346132 |
| MGI:7579358 | Plagl1it | pleiomorphic adenoma gene-like 1 intronic transcript | 10 | J:187821 / PMID:22723905 |
| MGI:104662 | Pml | promyelocytic leukemia | 9 | J:164563 |
| MGI:1933378 | Pole3 | polymerase (DNA directed), epsilon 3 (p17 subunit) | 4 | J:342607 |
| MGI:1917029 | Pphln1 | periphilin 1 | 15 | J:161428 |
| MGI:1913848 | Pphln1ps1 | periphilin 1, pseudogene 1 | 16 | J:161428 |
| MGI:1858214 | Ppm1d | protein phosphatase 1D magnesiumdependent | 11 | J:161428 |
| MGI:99655 | Prdm1 | PR domain containing 1, with ZNF domain | 10 | PMID: 38540967 |
| MGI:2682952 | Prdm10 | PR domain containing 10 | 9 | PMID: 31313299 |
| MGI:2448528 | Prdm13 | PR domain containing 13 | 4 | J:346132 |
| MGI:3588194 | Prdm14 | PR domain containing 14 | 1 | J:185718 / PMID:22482507 / PMID:24268575 /PMID:23670199 |
| MGI:1917923 | Prdm16 | PR domain containing 16 | 4 | J:269562 |
| MGI:1918029 | Prdm5 | PR domain containing 5 | 6 | J:60000 |
| MGI:2684938 | Prdm6 | PR domain containing 6 | 18 | J:346132 |
| MGI:1924880 | Prdm8 | PR domain containing 8 | 5 | J:346132 |
| MGI:2384854 | Prdm9 | PR domain containing 9 | 17 | J:346132 |
| MGI:3603756 | Primpol | primase and polymerase (DNA-directed) | 8 | J:164563 |
| MGI:2145955 | Prkaa1 | protein kinase, AMP-activated, alpha 1 catalytic subunit | 15 | J:346132 |
| MGI:1336173 | Prkaa2 | protein kinase, AMP-activated, alpha 2 catalytic subunit | 4 | J:346132 |
| MGI:97596 | Prkcb | protein kinase C, beta | 7 | J:346132 |
| MGI:104779 | Prkdc | protein kinase, DNA activated, catalytic polypeptide | 16 | J:346132 |
| MGI:97765 | Prm1 | protamine 1 | 16 | J:72247 |
| MGI:107846 | Prmt1 | protein arginine N-methyltransferase 1 | 7 | J:161428 |
| MGI:1316652 | Prmt2 | protein arginine N-methyltransferase 2 | 10 | J:161428 |
| MGI:1919224 | Prmt3 | protein arginine N-methyltransferase 3 | 7 | J:161428 |
| MGI:1351645 | Prmt5 | protein arginine N-methyltransferase 5 | 14 | J:164563 |
| MGI:2139971 | Prmt6 | protein arginine N-methyltransferase 6 | 3 | J:161428 |
| MGI:2384879 | Prmt7 | protein arginine N-methyltransferase 7 | 8 | J:161428 |
| MGI:3043083 | Prmt8 | protein arginine N-methyltransferase 8 | 6 | J:161428 |
| MGI:2142651 | Prmt9 | protein arginine methyltransferase 9 | 8 | J:161428 |
| MGI:2142116 | Psip1 | PC4 and SFRS1 interacting protein 1 | 4 | J:161428 |
| MGI:2143994 | Psme4 | proteasome (prosome, macropain) activator subunit 4 | 11 | J:197675 |
| MGI:97803 | Ptma | prothymosin alpha | 1 | J:161326 / PMID:20434447 |
| MGI:1918052 | Pwwp2a | PWWP domain containing 2A | 11 | J:164563 |
| MGI:2142008 | Pwwp2b | PWWP domain containing 2B | 7 | J:319689 |
| MGI:1915364 | Pwwp3a | PWWP domain containing 3A, DNA repair factor | 10 | J:161428 |
| MGI:1333807 | Rad17 | RAD17 checkpoint clamp loader component | 13 | J:346132 |
| MGI:108016 | Rad21 | RAD21 cohesin complex component | 15 | J:294078 / PMID:32328639 |
| MGI:109292 | Rad50 | RAD50 double strand break repair protein | 11 | J:164563 |
| MGI:3605986 | Rad54b | RAD54 homolog B (S. cerevisiae) | 4 | J:346132 |
| MGI:894697 | Rad54l | RAD54 like (S. cerevisiae) | 4 | J:346132 |
| MGI:1933196 | Rad54l2 | RAD54 like 2 (S. cerevisiae) | 9 | J:161428 |
| MGI:97848 | Rag1 | recombination activating 1 | 2 | J:60000 |
| MGI:97849 | Rag2 | recombination activating gene 2 | 2 | J:60000 |
| MGI:97874 | Rb1 | RB transcriptional corepressor 1 | 14 | J:164563 |
| MGI:1194912 | Rbbp4 | retinoblastoma binding protein 4 | 4 | J:87046 / PMID:14645126 |
| MGI:1918367 | Rbbp5 | retinoblastoma binding protein 5 | 1 | J:164563 |
| MGI:1194910 | Rbbp7 | retinoblastoma binding protein 7 | X | J:87046 / PMID:14645126 |
| MGI:103300 | Rbl1 | RB transcriptional corepressor like 1 | 2 | J:60000 |
| MGI:105085 | Rbl2 | RB transcriptional corepressor like 2 | 8 | J:60000 |
| MGI:1929092 | Rbm14 | RNA binding motif protein 14 | 19 | J:342587 |
| MGI:2443205 | Rbm15 | RNA binding motif protein 15 | 3 | J:164563 |
| MGI:1923598 | Rbm15b | RNA binding motif protein 15B | 9 | J:164563 |
| MGI:1918580 | Rcbtb1 | regulator of chromosome condensation (RCC1) and BTB (POZ) domain containing protein 1 | 14 | J:60000 |
| MGI:2444156 | Rccd1 | RCC1 domain containing 1 | 7 | J:60000 |
| MGI:106340 | Rcor1 | REST corepressor 1 | 12 | J:60000 |
| MGI:103290 | Rela | v-rel reticuloendotheliosis viral oncogene homolog A (avian) | 19 | J:272425 |
| MGI:1923627 | Reno1 | regulator of early neurogenesis 1 | 11 | J:301382 / PMID:32969152 |
| MGI:2683486 | Rere | arginine glutamic acid dipeptide (RE) repeats | 4 | J:87046 / PMID:14645126 |
| MGI:1914496 | Resf1 | retroelement silencing factor 1 | 6 | J:161428 |
| MGI:104897 | Rest | RE1-silencing transcription factor | 5 | J:155856 |
| MGI:1915315 | Rhno1 | RAD9-HUS1-RAD1 interacting nuclear orphan 1 | 6 | J:346132 |
| MGI:1922995 | Rian | RNA imprinted and accumulated in nucleus | 12 | J:290525 [PMID:26005002] |
| MGI:1098622 | Rif1 | replication timing regulatory factor 1 | 2 | J:213416 |
| MGI:1101770 | Ring1 | ring finger protein 1 | 17 | J:104662 / PMID:16359901 / PMID:15525528 |
| MGI:1919202 | Riox1 | ribosomal oxygenase 1 | 12 | J:90013 / PMID:14742713 |
| MGI:1914264 | Riox2 | ribosomal oxygenase 2 | 16 | J:346132 |
| MGI:1924705 | Rlf | rearranged L-myc fusion sequence | 4 | J:225652 / PMID:25857663 |
| MGI:1342291 | Rlim | ring finger protein | X | J:212497 / PMID:24613346 |
| MGI:1917488 | Rnf168 | ring finger protein 168 | 16 | J:164563 |
| MGI:1101759 | Rnf2 | ring finger protein 2 | 1 | J:104662 / PMID:16359901 / PMID:15525528 |
| MGI:1925927 | Rnf20 | ring finger protein 20 | 4 | J:346132 |
| MGI:2142048 | Rnf40 | ring finger protein 40 | 7 | J:60000 |
| MGI:1929069 | Rnf8 | ring finger protein 8 | 17 | J:164563 |
| MGI:1930076 | Rps6ka4 | ribosomal protein S6 kinase, polypeptide 4 | 19 | J:346132 |
| MGI:1920336 | Rps6ka5 | ribosomal protein S6 kinase, polypeptide 5 | 12 | J:346132 |
| MGI:1914251 | Rrp8 | ribosomal RNA processing 8 | 7 | J:161428 |
| MGI:2444993 | Rsbn1 | rosbin, round spermatid basic protein 1 | 3 | J:346132 |
| MGI:2682305 | Rsf1 | remodeling and spacing factor 1 | 7 | J:164563 |
| MGI:3044162 | Rsl1 | regulator of sex limited protein 1 | 13 | J:188793 / PMID:22801370 |
| MGI:1309480 | Rtf1 | RTF1, Paf1/RNA polymerase II complex component | 2 | J:149841 |
| MGI:1928760 | Ruvbl1 | RuvB-like AAA ATPase 1 | 6 | J:161428 |
| MGI:1342299 | Ruvbl2 | RuvB-like AAA ATPase 2 | 7 | J:161428 |
| MGI:1929059 | Rybp | RING1 and YY1 binding protein | 6 | J:164563 |
| MGI:3648043 | Rybp-ps | RING1 and YY1 binding protein, pseudogene | 13 | J:164563 |
| MGI:1098658 | Sa1 | STAG1 cohesin complex component | 9 | PMID: 31495782 |
| MGI:1098583 | Sa2 | STAG2 cohesin complex component | X | PMID: 31495782 |
| MGI:2142433 | Samd1 | sterile alpha motif domain containing 1 | 8 | J:60000 |
| MGI:1923203 | Samd7 | sterile alpha motif domain containing 7 | 3 | J:253631 |
| MGI:1858230 | Sart3 | squamous cell carcinoma antigen recognized by T cells 3 | 5 | J:164563 |
| MGI:105084 | Satb1 | special AT-rich sequence binding protein 1 | 17 | J:83186 / PMID:12692553 / PMID:21930775 |
| MGI:2679336 | Satb2 | special AT-rich sequence binding protein 2 | 1 | J:135318 / PMID:18255031 |
| MGI:104574 | Scc1 | protein tyrosine phosphatase receptor type J | 2 | PMID: 32328639 |
| MGI:1352762 | Scmh1 | Scm polycomb group homolog 1 | 4 | J:119909 [PMID:17215307] |
| MGI:2668443 | Sdr16c5 | short chain dehydrogenase/reductase family 16C | 4 | J:164563 |
| MGI:1927947 | Selenof | selenoprotein F | 3 | J:155856 |
| MGI:1860267 | Set | SET nuclear oncogene | 2 | J:72247 |
| MGI:1933199 | Setbp1 | SET binding protein 1 | 18 | J:346132 |
| MGI:2446244 | Setd1a | SET domain containing 1A | 7 | J:164563 |
| MGI:2652820 | Setd1b | SET domain containing 1B | 5 | J:346132 |
| MGI:1918177 | Setd2 | SET domain containing 2 | 9 | J:164563 |
| MGI:1289184 | Setd3 | SET domain containing 3 | 12 | J:346132 |
| MGI:2136890 | Setd4 | SET domain containing 4 | 16 | J:161428 |
| MGI:1920145 | Setd5 | SET domain containing 5 | 6 | J:346132 |
| MGI:1913333 | Setd6 | SET domain containing 6 | 8 | PMID: 21131967 |
| MGI:1920501 | Setd7 | SET domain containing (lysine methyltransferase) 7 | 3 | J:194231 / PMID:23509280 |
| MGI:1915206 | Setd8 | lysine methyltransferase 5A | 5 | PMID: 32178723 |
| MGI:1934229 | Setdb1 | SET domain | 3 | J:161428 |
| MGI:2685139 | Setdb2 | SET domain | 14 | J:161428 |
| MGI:1921979 | Setmar | SET domain without mariner transposase fusion | 6 | J:346132 |
| MGI:1932339 | Sf3b1 | splicing factor 3b, subunit 1 | 1 | J:320057 |
| MGI:1859609 | Sfmbt1 | Scm-like with four mbt domains 1 | 14 | J:60000 |
| MGI:1918764 | Sfpq | splicing factor proline/glutamine rich (polypyrimidine tract binding protein associated) | 4 | J:173532 |
| MGI:1922815 | Sgf29 | SAGA complex associated factor 29 | 7 | J:164563 |
| MGI:1917581 | Shprh | SNF2 histone linker PHD RING helicase | 10 | J:72247 |
| MGI:107157 | Sin3a | transcriptional regulator | 9 | J:164563 |
| MGI:2135607 | Sirt1 | sirtuin 1 | 10 | J:197487 / PMID:23160044 |
| MGI:1927664 | Sirt2 | sirtuin 2 | 7 | J:162081 / PMID:20562830 |
| MGI:1927665 | Sirt3 | sirtuin 3 | 7 | J:346132 |
| MGI:1922637 | Sirt4 | sirtuin 4 | 5 | J:346132 |
| MGI:1915596 | Sirt5 | sirtuin 5 | 13 | J:161428 |
| MGI:1354161 | Sirt6 | sirtuin 6 | 10 | J:164563 |
| MGI:2385849 | Sirt7 | sirtuin 7 | 11 | J:161428 |
| MGI:103575 | Skp1 | S-phase kinase-associated protein 1 | 11 | J:164563 |
| MGI:103241 | Slk | STE20-like kinase | 19 | J:346132 |
| MGI:99543 | Slx | Sycp3 like X-linked | X | J:164563 |
| MGI:2687328 | Sly | Sycp3 like Y-linked | Y | J:164563 |
| MGI:1935127 | Smarca1 | SNF2 related chromatin remodeling ATPase 1 | X | J:164563 |
| MGI:99603 | Smarca2 | SWI/SNF related BAF chromatin remodeling complex subunit ATPase 2 | 19 | J:69532 / PMID:11318604 / PMID:11163203 |
| MGI:88192 | Smarca4 | SWI/SNF related | 9 | J:103896 / PMID:16287714 / PMID:11163203 / PMID:34910916 |
| MGI:1935129 | Smarca5 | SNF2 related chromatin remodeling ATPase 5 | 8 | J:76488 / PMID:11980720 / PMID:11980720 / PMID:11532953 |
| MGI:95453 | Smarcad1 | SNF2 related chromatin remodeling ATPase with DExD box 1 | 6 | J:161428 |
| MGI:1859183 | Smarcal1 | SWI/SNF related matrix associated, actin dependent regulator of chromatin, subfamily a-like 1 | 1 | J:346132 |
| MGI:1328366 | Smarcb1 | SWI/SNF related BAF chromatin remodeling complex subunit B1 | 10 | J:161428 |
| MGI:1203524 | Smarcc1 | SWI/SNF related BAF chromatin remodeling complex subunit C1 | 9 | J:164563 |
| MGI:1915344 | Smarcc2 | SWI/SNF related BAF chromatin remodeling complex subunit C2 | 10 | J:164563 |
| MGI:1933623 | Smarcd1 | SWI/SNF related BAF chromatin remodeling complex subunit D1 | 15 | J:164563 |
| MGI:1933621 | Smarcd2 | SWI/SNF related BAF chromatin remodeling complex subunit D2 | 11 | J:164563 |
| MGI:1914243 | Smarcd3 | SWI/SNF related BAF chromatin remodeling complex subunit D3 | 5 | J:164563 |
| MGI:1927347 | Smarce1 | SWI/SNF related BAF chromatin remodeling complex subunit E1 | 11 | J:164563 |
| MGI:7286091 | Smc1 | serum myostatin concentration 1 | 3 | PMID: 31331360 |
| MGI:1339795 | Smc3 | structural maintenance of chromosomes 3 | 19 | PMID: 31331360 |
| MGI:1921605 | Smchd1 | SMC hinge domain containing 1 | 17 | J:204566 [PMID:23754746 / PMID:18425126 / PMID:18425126 |
| MGI:104790 | Smyd1 | SET and MYND domain containing 1 | 6 | J:76212 / PMID:11923873 |
| MGI:1915889 | Smyd2 | SET and MYND domain containing 2 | 1 | J:346132 |
| MGI:1916976 | Smyd3 | SET and MYND domain containing 3 | 1 | J:199171 / PMID:23752591 |
| MGI:108048 | Smyd5 | SET and MYND domain containing 5 | 6 | J:161428 |
| MGI:98330 | Snai1 | snail family zinc finger 1 | 2 | J:206163 |
| MGI:1096393 | Snai2 | snail family zinc finger 2 | 16 | J:130039 / PMID:17905753 |
| MGI:1915099 | Sororin | cell division cycle associated 5(cdca5) | 11 | PMID: 21059905 |
| MGI:98371 | Sox9 | SRY (sex determining region Y)-box 9 | 11 | J:164563 |
| MGI:1861380 | Sphk2 | sphingosine kinase 2 | 7 | J:164563 |
| MGI:98282 | Spi1 | Spi-1 proto-oncogene | 2 | J:164563 |
| MGI:109242 | Spin1 | spindlin 1 | 13 | J:60000 |
| MGI:3652045 | Spocd1 | SPOC domain containing 1 | 4 | J:295943 |
| MGI:2142062 | Spty2d1 | SPT2 chromatin protein domain containing 1 | 7 | J:161428 |
| MGI:2444036 | Srcap | Snf2-related CREBBP activator protein | 7 | J:161428 |
| MGI:1201408 | Srpk2 | serine/arginine-rich protein specific kinase 2 | 5 | J:164563 |
| MGI:107708 | Ss18 | SS18, subunit of BAF chromatin remodeling complex | 18 | J:283189 |
| MGI:2444061 | Ss18l1 | SS18, nBAF chromatin remodeling complex subunit like 1 | 2 | J:60000 |
| MGI:107912 | Ssrp1 | structure specific recognition protein 1 | 2 | J:164563 |
| MGI:1098658 | Stag1 | STAG1 cohesin complex component | 9 | PMID: 40118039 |
| MGI:1098583 | Stag2 | STAG2 cohesin complex component | X | PMID: 40118039 |
| MGI:1926056 | Stpg1 | sperm tail PG rich repeat containing 1 | 4 | J:345476 |
| MGI:1922717 | Stpg4 | sperm tail PG rich repeat containing 4 | 17 | J:161428 |
| MGI:1919204 | Suds3 | suppressor of defective silencing 3 homolog (S. cerevisiae) | 5 | J:164563 |
| MGI:1890948 | Supt16 | SPT16 | 14 | J:164563 |
| MGI:107416 | Supt4a | SPT4A, DSIF elongation factor subunit | 11 | J:72247 |
| MGI:1335090 | Supt4b | SPT4B, DSIF elongation factor subunit | 10 | J:72247 |
| MGI:1202400 | Supt5 | suppressor of Ty 5, DSIF elongation factor subunit | 7 | J:72247 |
| MGI:107726 | Supt6 | SPT6 | 11 | J:161428 |
| MGI:1099440 | Suv39h1 | suppressor of variegation 3-9 1 | X | J:164563 |
| MGI:1890396 | Suv39h2 | suppressor of variegation 3-9 2 | 2 | J:164563 |
| MGI:2444557 | Suv420h1 | lysine methyltransferase 5B | 19 | PMID: 38052811 |
| MGI:2385262 | Suv420h2 | lysine methyltransferase 5C | 7 | PMID: 23599346 |
| MGI:1261758 | Suz12 | SUZ12 polycomb repressive complex 2 subunit | 11 | J:104662 / PMID:16359901 / PMID:27892467 |
| MGI:105931 | Sycp1 | synaptonemal complex protein 1 | 3 | J:182264 / PMID:22164254 |
| MGI:109542 | Sycp3 | synaptonemal complex protein 3 | 10 | J:182264 / PMID:22164254 |
| MGI:2144471 | Tada2a | transcriptional adaptor 2A | 11 | J:162081 / PMID:20562830 |
| MGI:3035274 | Tada2b | transcriptional adaptor 2B | 5 | J:161428 |
| MGI:1915724 | Tada3 | transcriptional adaptor 3 | 6 | J:162081 / PMID:20562830 |
| MGI:1336878 | Taf1 | TATA-box binding protein associated factor 1 | X | J:346132 |
| MGI:1346320 | Taf10 | TATA-box binding protein associated factor 10 | 7 | J:346132 |
| MGI:98480 | Tal1 | T cell acute lymphocytic leukemia 1 | 4 | J:164563 |
| MGI:1921694 | Tasor | transcription activation suppressor | 14 | J:161428 |
| MGI:2145274 | Tasor2 | transcription activation suppressor family member 2 | 13 | J:72247 |
| MGI:2441730 | Tbl1xr1 | transducin (beta)-like 1X-linked receptor 1 | 3 | J:60000 |
| MGI:107404 | Tbr1 | T-box brain transcription factor 1 | 2 | J:266522 [PMID:30186101] |
| MGI:98510 | Tcf3 | transcription factor 3 | 10 | J:105503 [PMID:16428437] |
| MGI:108247 | Tdg | thymine DNA glycosylase | 10 | J:174772 |
| MGI:1933218 | Tdrd1 | tudor domain containing 1 | 19 | J:152108 |
| MGI:1919231 | Tdrd12 | tudor domain containing 12 | 7 | J:202012 |
| MGI:2444023 | Tdrd3 | tudor domain containing 3 | 14 | J:60000 |
| MGI:2684949 | Tdrd5 | tudor domain containing 5 | 1 | J:170319 |
| MGI:1921941 | Tdrd9 | tudor domain containing 9 | 12 | J:238859 [PMID:27473657] |
| MGI:1098693 | Tet1 | tet methylcytosine dioxygenase 1 | 10 | J:207929 / PMID:24291790 / PMID:20639862 / PMID:24291790 |
| MGI:2443298 | Tet2 | tet methylcytosine dioxygenase 2 | 3 | J:178415 / PMID:21803851 / PMID:20639862 |
| MGI:2446229 | Tet3 | tet methylcytosine dioxygenase 3 | 6 | J:163464 / PMID:20639862 / PMID:23690950 |
| MGI:1934816 | Tex15 | testis expressed gene 15 meiosis and synapsis associated | 8 | J:288732 |
| MGI:1920929 | Tex19.1 | testis expressed gene 19.1 | 11 | J:159793 / PMID:18802469 |
| MGI:1918206 | Tex19.2 | testis expressed gene 19.2 | 11 | J:164563 |
| MGI:106032 | Tfap2c | transcription factor AP-2 | 2 | J:164563 |
| MGI:1916964 | Tfpt | TCF3 (E2A) fusion partner | 7 | J:164563 |
| MGI:98731 | Tgm2 | transglutaminase 2, C polypeptide | 2 | J:346132 |
| MGI:1916259 | Thap7 | THAP domain containing 7 | 16 | J:164563 |
| MGI:2441683 | Tlk1 | tousled-like kinase 1 | 2 | J:164563 |
| MGI:1346023 | Tlk2 | tousled-like kinase 2 (Arabidopsis) | 11 | J:164563 |
| MGI:2685210 | Tm9sf5 | transmembrane 9 superfamily member 5 | X | J:320690 |
| MGI:98784 | Tnp1 | transition protein 1 | 1 | J:161428 |
| MGI:98785 | Tnp2 | transition protein 2 | 16 | J:107120 |
| MGI:1919999 | Tonsl | tonsoku-like, DNA repair protein | 15 | J:60000 |
| MGI:98788 | Top1 | topoisomerase (DNA) I | 2 | J:164563 |
| MGI:1920018 | Topbp1 | topoisomerase (DNA) II binding protein 1 | 9 | J:346132 |
| MGI:2181659 | Tox | thymocyte selection-associated high mobility group box | 4 | J:60000 |
| MGI:1922066 | Tpr | translocated promoter region | 1 | J:164563 |
| MGI:97904 | Trim27 | tripartite motif-containing 27 | 13 | J:66240 / PMID:10976108 |
| MGI:109274 | Trim28 | tripartite motif-containing 28 | 7 | J:161428 |
| MGI:2153072 | Trim37 | tripartite motif-containing 37 | 11 | J:164563 |
| MGI:1309481 | Trip12 | thyroid hormone receptor interactor 12 | 1 | J:164563 |
| MGI:1914924 | Trmt112 | tRNA methyltransferase 11-2 | 19 | J:164563 |
| MGI:98834 | Trp53 | transformation related protein 53 | 11 | J:342605 |
| MGI:1351320 | Trp53bp1 | transformation related protein 53 binding protein 1 | 2 | J:346132 |
| MGI:1330810 | Trp63 | transformation related protein 63 | 16 | J:177729 [PMID:21930775] |
| MGI:2153272 | Trrap | transformation/transcription domainassociated protein | 5 | J:60000 |
| MGI:1336196 | Tsix | X (inactive)-specific transcript | X | J:99399 / PMID:15992549 / PMID:24268575 / PMID:15992549 |
| MGI:1298395 | Tspyl1 | testis-specific protein, Y-encoded-like 1 | 10 | J:72247 |
| MGI:106244 | Tspyl2 | TSPY-like 2 | X | J:72247 |
| MGI:106393 | Tspyl4 | TSPY-like 4 | 10 | J:72247 |
| MGI:2442458 | Tspyl5 | testis-specific protein, Y-encoded-like 5 | 15 | J:72247 |
| MGI:2148775 | Tssk6 | testis-specific serine kinase 6 | 8 | J:98891 |
| MGI:3651956 | Ttc39aos1 | Ttc39a opposite strand RNA 1 | 4 | J:234649 / PMID:27315481 |
| MGI:105044 | Ttf1 | transcription termination factor | 2 | J:38595 / PMID:9049305 |
| MGI:1921294 | Ttf2 | transcription termination factor, RNA polymerase II | 3 | J:346132 |
| MGI:1922675 | Tti1 | TELO2 interacting protein 1 | 2 | J:345476 |
| MGI:2445126 | Tut4 | terminal uridylyl transferase 4 | 4 | J:164563 |
| MGI:2387179 | Tut7 | terminal uridylyl transferase 7 | 13 | J:164563 |
| MGI:102959 | Ube2a | ubiquitin-conjugating enzyme E2A | X | J:164563 |
| MGI:102944 | Ube2b | ubiquitin-conjugating enzyme E2B | 11 | J:124051 / PMID:17488778 |
| MGI:1891307 | Ubn1 | ubinuclein 1 | 16 | J:161428 |
| MGI:2444236 | Ubn2 | ubinuclein 2 | 6 | J:161428 |
| MGI:1861099 | Ubr2 | ubiquitin protein ligase E3 component nrecognin 2 | 17 | J:157596 |
| MGI:1918040 | Ubr5 | ubiquitin protein ligase E3 component nrecognin 5 | 15 | J:164563 |
| MGI:98512 | Ubtf | upstream binding transcription factor | 11 | J:146000 / PMID:19103806 |
| MGI:1914848 | Uchl5 | ubiquitin carboxyl-terminal esterase L5 | 1 | J:164563 |
| MGI:1338889 | Uhrf1 | ubiquitin-like | 17 | J:164563 |
| MGI:1923718 | Uhrf2 | ubiquitin-like | 19 | J:161428 |
| MGI:103185 | Uimc1 | ubiquitin interaction motif containing 1 | 13 | J:164563 |
| MGI:107995 | Upf1 | UPF1 RNA helicase and ATPase | 8 | J:116443 / PMID:16789828 |
| MGI:1914281 | Upf3a | UPF3 regulator of nonsense transcripts homolog A (yeast) | 8 | J:116443 / PMID:16789828 |
| MGI:1915384 | Upf3b | UPF3 regulator of nonsense transcripts homolog B (yeast) | X | J:116443 / PMID:16789828 |
| MGI:101857 | Usp15 | ubiquitin specific peptidase 15 | 10 | J:161428 |
| MGI:1921362 | Usp16 | ubiquitin specific peptidase 16 | 16 | J:346132 |
| MGI:1353665 | Usp21 | ubiquitin specific peptidase 21 | 1 | J:148656 |
| MGI:2144157 | Usp22 | ubiquitin specific peptidase 22 | 11 | J:346132 |
| MGI:2152450 | Usp3 | ubiquitin specific peptidase 3 | 9 | J:164563 |
| MGI:1919594 | Usp36 | ubiquitin specific peptidase 36 | 11 | J:164563 |
| MGI:2685391 | Usp49 | ubiquitin specific peptidase 49 | 17 | J:346132 |
| MGI:3588217 | Usp51 | ubiquitin specific protease 51 | X | J:164563 |
| MGI:2182061 | Usp7 | ubiquitin specific peptidase 7 | 16 | J:164563 |
| MGI:1919230 | Utp3 | UTP3 small subunit processome component | 5 | J:60000 |
| MGI:1095419 | Utx | lysine (K)-specific demethylase 6A | X | PMID: 22192413 |
| MGI:894810 | Uty | ubiquitously transcribed tetratricopeptide repeat containing | Y | J:180125 / PMID:22192413 |
| MGI:2684917 | Vcpkmt | valosin containing protein lysine (K) methyltransferase | 12 | J:346132 |
| MGI:1202305 | Vps72 | vacuolar protein sorting 72 | 3 | J:164563 |
| MGI:1261847 | Vrk1 | vaccinia related kinase 1 | 12 | J:164563 |
| MGI:2387357 | Wac | WW domain containing adaptor with coiledcoil | 18 | J:164563 |
| MGI:2675859 | Wapl | WAPL cohesin release factor | 14 | PMID: 23975099 |
| MGI:104709 | Wbp2 | WW domain binding protein 2 | 11 | J:164563 |
| MGI:2443514 | Wdhd1 | WD repeat and HMG-box DNA binding protein 1 | 14 | J:182876 / PMID:21266480 |
| MGI:2155884 | Wdr5 | WD repeat domain 5 | 2 | J:164563 |
| MGI:1332638 | Wiz | widely-interspaced zinc finger motifs17 | 19 | PMID: 32294452 |
| MGI:98968 | Wt1 | WT1 transcription factor | 2 | J:164563 |
| MGI:98974 | Xist | inactive X specific transcripts | X | J:99399 / PMID:15992549 / PMID:24268575 / PMID:15992549 / PMID:23658530 |
| MGI:98976 | Xlr | X-linked lymphocyte-regulated | X | J:164563 |
| MGI:2447762 | Yeats2 | YEATS domain containing 2 | 16 | J:161428 |
| MGI:1927224 | Yeats4 | YEATS domain containing 4 | 10 | J:161428 |
| MGI:2443713 | Ythdc1 | YTH domain containing 1 | 5 | J:164563 |
| MGI:99150 | Yy1 | YY1 transcription factor | 12 | J:164563 |
| MGI:2442326 | Zbtb1 | zinc finger and BTB domain containing 1 | 12 | J:164563 |
| MGI:1335091 | Zbtb7a | zinc finger and BTB domain containing 7a | 10 | J:164563 |
| MGI:2685899 | Zcwpw1 | zinc finger, CW type with PWWP domain 1 | 5 | J:346132 |
| MGI:1921134 | Zdbf2 | zinc finger | 1 | J:261327 / PMID:27841881 |
| MGI:1890378 | Zfp110 | zinc finger protein 110 | 7 | J:164563 |
| MGI:3036278 | Zfp273 | zinc finger protein 273 | 13 | J:164563 |
| MGI:2682313 | Zfp335 | zinc finger protein 335 | 2 | J:164563 |
| MGI:2176229 | Zfp369 | zinc finger protein 369 | 13 | J:164563 |
| MGI:99187 | Zfp42 | zinc finger protein 42 | 8 | J:169234 / PMID:21233130 |
| MGI:2143340 | Zfp445 | zinc finger protein 445 | 9 | J:164563 |
| MGI:107690 | Zfp462 | zinc finger protein 462 | 4 | J:158807 / PMID:20219459 |
| MGI:1919922 | Zfp518a | zinc finger protein 518A | 19 | J:60000 |
| MGI:2140750 | Zfp518b | zinc finger protein 518B | 5 | J:60000 |
| MGI:99204 | Zfp57 | zinc finger protein 57 | 17 | J:302583 / PMID:33500348 |
| MGI:3040674 | Zfp708 | zinc finger protein 708 | 13 | J:164563 |
| MGI:2444807 | Zfp827 | zinc finger protein 827 | 8 | J:164563 |
| MGI:99213 | Zfy2 | zinc finger protein 2 | Y | J:239332 / PMID:27742779 |
| MGI:1890508 | Zmpste24 | zinc metallopeptidase STE24 | 4 | J:134546 / PMID:16079796 / PMID:20961378 |
| MGI:1913755 | Zmynd11 | zinc finger, MYND domain containing 11 | 13 | J:60000 |
| MGI:2138982 | Znfx1 | Zinc Finger NFX1-Type Containing 1 | 2 | J:161428 |
| MGI:1917353 | Znhit1 | zinc finger, HIT domain containing 1 | 5 | J:256297 |
| MGI:1918362 | Zranb3 | zinc finger, RAN-binding domain containing 3 | 1 | J:346132 |
| MGI:2444286 | Zzef1 | zinc finger, ZZ-type with EF hand domain 1 | 11 | J:346132 |
| MGI:1920453 | Zzz3 | Zinc Finger ZZ-Type Containing 3 | 3 | J:164563 |

**Table 1. List of chromatin structural factors.**
