# Supplementary material for: Chromatin structural gene expression stratifies cardiac cell populations in health and disease
Source: Epigenetics. 2025 Oct 21;20(1):2566505. doi: 10.1080/15592294.2025.2566505 (PMC12542603; doi:10.1080/15592294.2025.2566505)
Supplement: Supplemental Material [file KEPI_A_2566505_SM2805.zip › SI-tables/Table_2.docx]

| **Gene** | **Specie** | **Primers** |
| --- | --- | --- |
| HMGN1 | Human | GCGAAGCCGAAAAAGGCAG  TCCGCAGGTAAGTCTTCTTTAGT |
| HMGN2 | Human | AGTTTGGCTTGGAATGCTGC  AGCAGAACGTACCCTGTTCC |
| HMGN3 | Human | GAGCCCACAAGACGGTCTG  TCTTCCCTTTAGCACCTCTGC |
| HMGN4 | Human | GATCAGCTCGGTTGTCTGCTA  GCAGGGTTGTTCCCATCCTT |
| HMGN5 | Human | CAGGTCAAGGTGATATGAGGCA  GCTTGGGCACTTGTATCTATGT |
| GAPDH | Human | TCTTCCCTTTAGCACCTCTGC  GGCTGTTGTCATACTTCTCATGG |
| α–Actinin | Human | TCCATCGGAGCCGAAGAAATC  GTGTCGGTGGATCAAAGCACA |
| Vimentin | Human | GACGCCATCAACACCGAGTT  CTTTGTCGTTGGTTAGCTGGT |

**Table 2. Primers used for qPCR.**

**Table 2. Primers used for qPCR.**
