# Supplementary material for: Chromatin structural gene expression stratifies cardiac cell populations in health and disease
Source: Epigenetics. 2025 Oct 21;20(1):2566505. doi: 10.1080/15592294.2025.2566505 (PMC12542603; doi:10.1080/15592294.2025.2566505)
Supplement: Supplemental Material [file KEPI_A_2566505_SM7732.zip › SI-figures/Supplementary Figures caption.docx]

**Supplementary Figure 1. Reanalysis of human snRNA-seq data confirms previously reported findings.** Unsupervised UMAP clustering of the dataset from Koenig et al.^11^ reproduces the original clustering patterns observed between healthy donors and DCM patients.

**Supplementary Figure 2. (A)** Characteristic marker genes of each identified cell population. **(B)** Gene ontology enrichment scatterplot showing the most relevant terms with the lowest p-values for each cell type. **(C)** Summary of sequencing quality metrics for cardiomyocytes (CM), fibroblasts (FB), endothelial cells (EC), smooth muscle cells (SMC), macrophages (MP), T cells (TC), and neurons (N). For each cell type, the table shows the total number of cells captured, the total number of unique genes detected across all cells, and the median number of genes detected per cell after quality filtering. These metrics reflect both the sequencing depth and transcriptional complexity of each cell population.

**Supplementary Figure 3. Demographic characteristics of human subjects included in the snRNA-seq dataset.** Gender, race, and age information of the human subjects from the snRNA-seq dataset generated and published by Koenig et al., which was used in the analyses presented in this manuscript. AA = African American; W = White.

**Supplementary Figure 4. Percentage of cells assigned to each cluster identified by Unsupervised Uniform Manifold Approximation and Projection (UMAP) analysis.** Clusters include cardiomyocytes (CM), fibroblasts (FB), endothelial cells (EC), smooth muscle cells (SMC), macrophages (MP), T cells (TC).

**Supplementary Figure 5. Cardiomyocyte marker expression and fibroblast contamination assessment. (A)** Immunofluorescence staining of isolated cardiomyocytes showing the cardiomyocyte marker Troponin T (green) with a higher magnification inset of a single cell displaying organized sarcomeric structure. Scale bar: 40µm. **(B)** Quantitative RT-PCR analysis of HMGN3, Vimentin (fibroblast marker) and Actinin (cardiomyocyte marker) mRNA expression. GAPDH was used to normalize cDNA input, and HMGN3 served as an internal reference to evaluate relative expression levels of Vimentin and Actinin. Data are presented as mean ± SEM, p < 0.01 (**), unpaired two-tailed t-test. **(C)** Immunofluorescence images showing α-Actinin (red) and nuclear DAPI staining (blue) in isolated cardiomyocytes. Merged images illustrate the organized sarcomeric arrangement of α-Actinin surrounding centrally located nuclei. Scale bar: 20 µm.

**Supplementary Figure 6. Human subject clinical data.** Available data on patients from whom heart samples were obtained is provided.

**Supplementary Figure 7. Echocardiographic measurements after TAC surgery.** Eleven weeks after TAC surgery, treated mice (red) show a significant decrease in ejection fraction (EF) and a significant increase in left ventricular internal dimension during diastole (LVIDd) and systole (LVIDs) compared to SHAM animals (green). Lines indicate the mean value for each parameter, and error bars represent the standard deviation (n = 10 mice per group).

**Supplementary Figure 8. Transcriptomic changes upon HMGN3 knockdown. (A)** PCA plot showing clustering of transcriptomic profiles from scramble control (blue) and siHMGN3 (red) samples, indicating distinct separation between conditions. **(B)** Volcano plot displaying differential gene expression between scramble and siHMGN3 conditions. The x-axis represents log₂ fold change and the y-axis represents –log₁₀(p-value). Significantly upregulated and downregulated genes are highlighted in orange, with selected genes labeled.

**Supplementary Figure 9. HMGN3 depletion reduces H3K27ac levels and alters regulatory element activity. (A)** Western blot analysis (top) and corresponding quantification (bottom) demonstrate a reduction in global levels of the active histone marks H3K27ac in AC16 cells 72 hours after HMGN3 knockdown. (*p < 0.05). **(B)** Motif enrichment analysis of differential H3K27ac regions. Shown are transcription factor motifs enriched in regions with decreased (blue) or increased (red) H3K27ac after siHMGN3. The x-axis represents the percentage of sequences above background, and the y-axis represents significance (–log₁₀ FDR). **(C)** Genomic distribution of differential H3K27ac peaks associated with genes identified as differentially expressed between the scramble and siHMGN3 groups. Pie charts display the proportions of peaks located in promoters, exons, introns, intergenic regions, or other genomic regions for sites losing (left) or gaining (right) H3K27ac after HMGN3 knockdown. Peaks gaining H3K27ac in upregulated genes are predominantly found in regulatory regions (introns and intergenic regions), while peaks losing H3K27ac in downregulated genes are enriched at promoters.
