# Supplementary figures and images for: Chromatin structural gene expression stratifies cardiac cell populations in health and disease
Source: Epigenetics. 2025 Oct 21;20(1):2566505. doi: 10.1080/15592294.2025.2566505 (PMC12542603; doi:10.1080/15592294.2025.2566505)

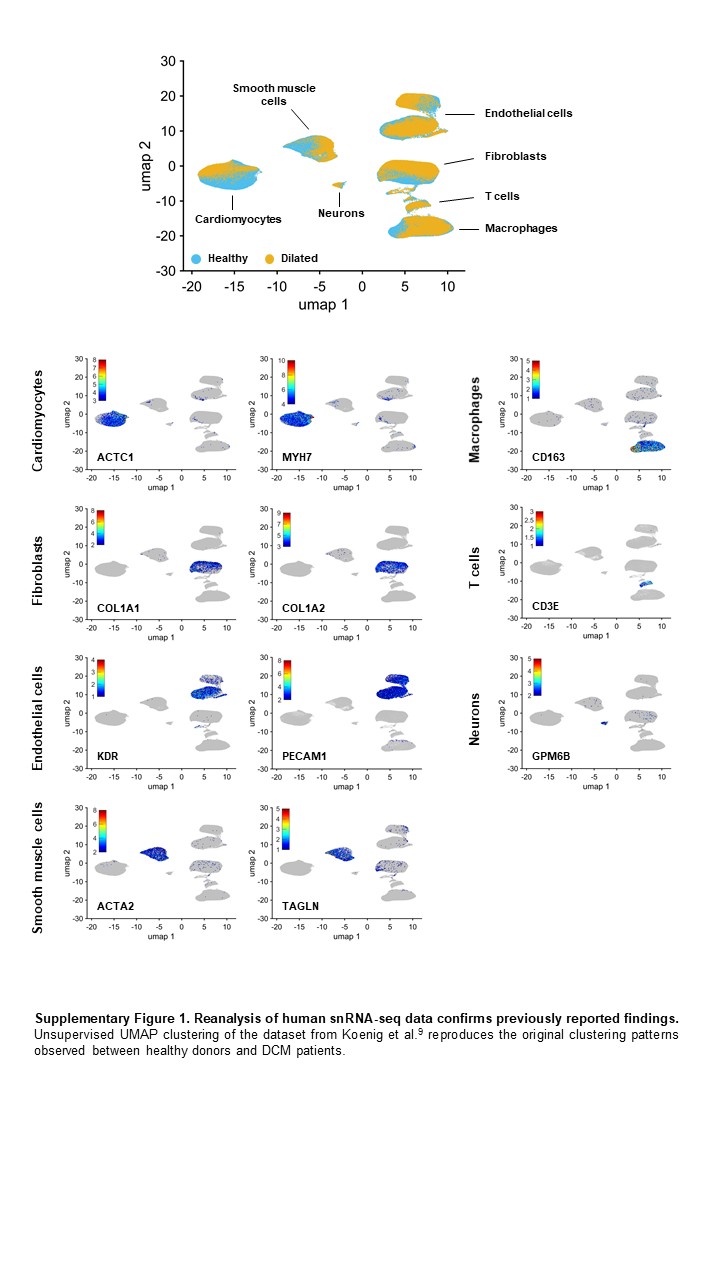

Supplement: Supplemental Material [file KEPI_A_2566505_SM7732.zip › SI-figures/Sup._Fig.1.JPG]

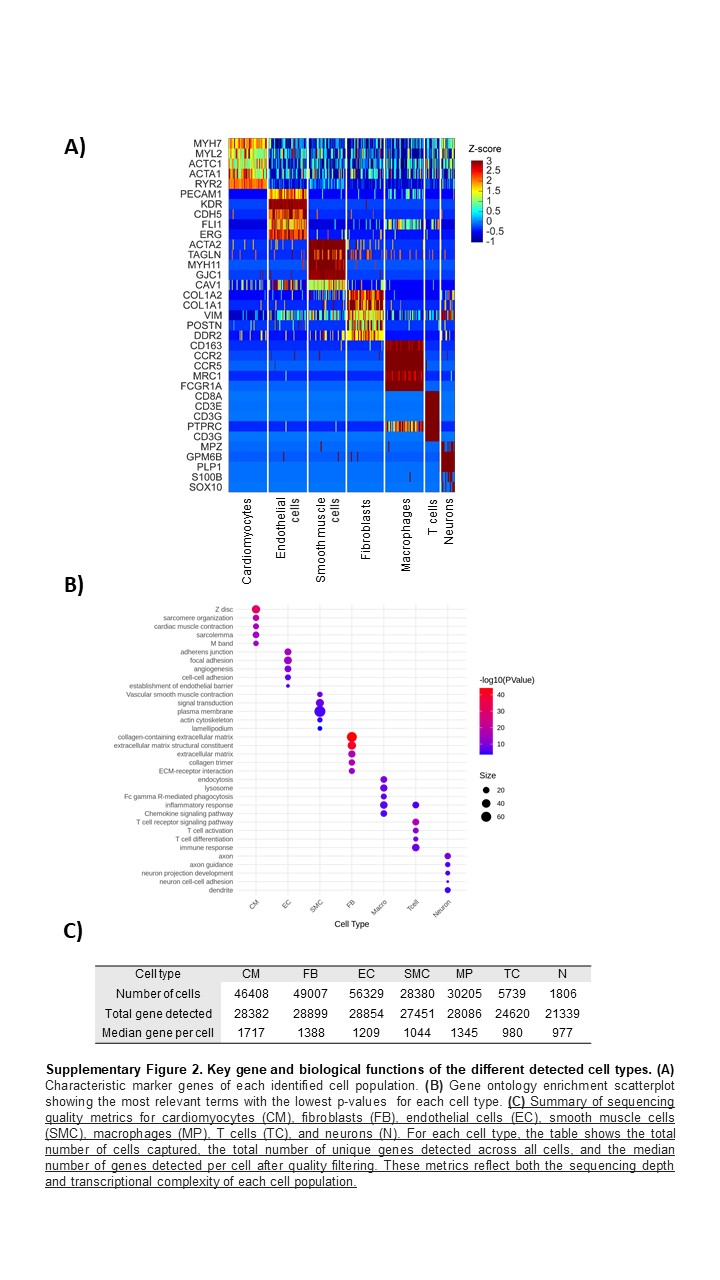

Supplement: Supplemental Material [file KEPI_A_2566505_SM7732.zip › SI-figures/Sup._Fig.2.JPG]

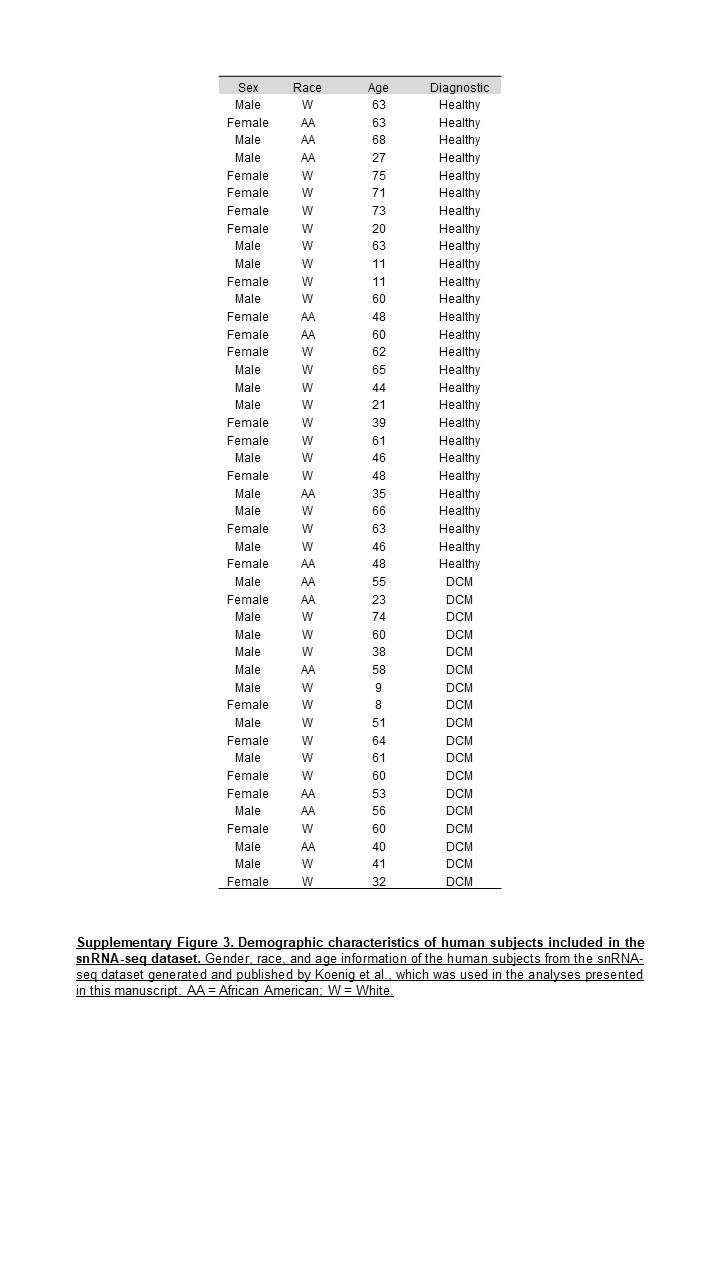

Supplement: Supplemental Material [file KEPI_A_2566505_SM7732.zip › SI-figures/Sup._Fig.3.JPG]

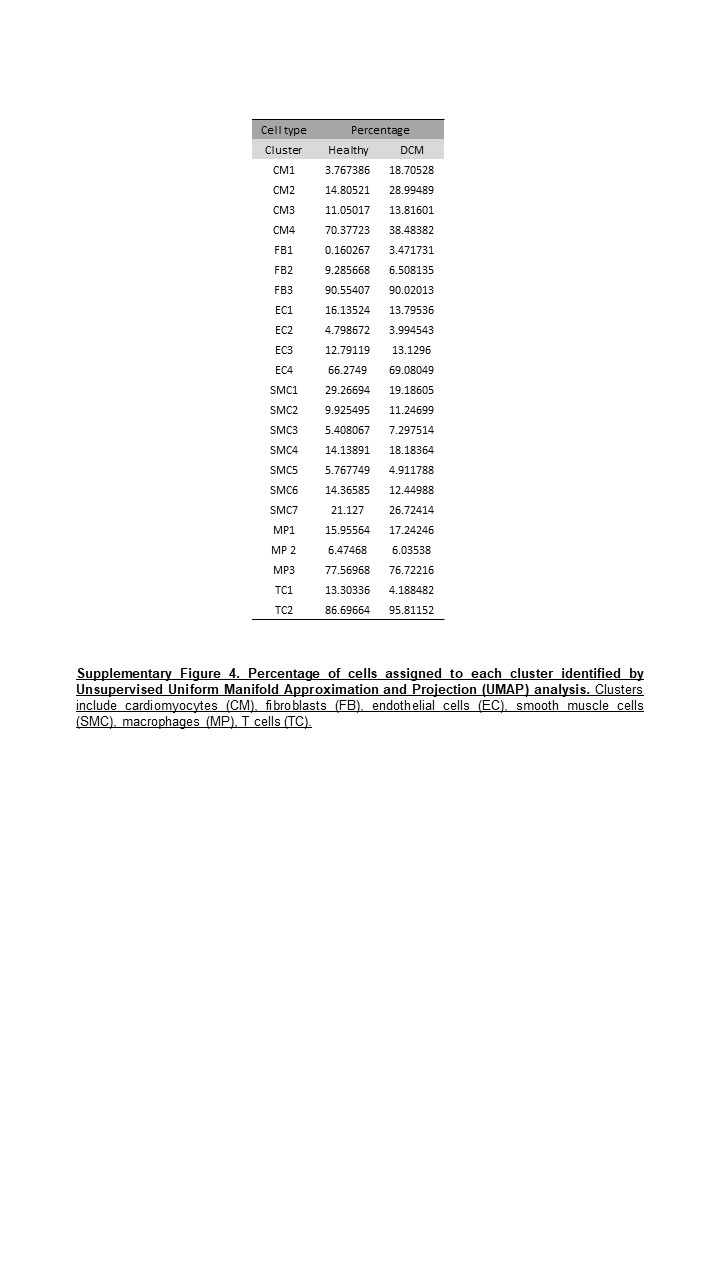

Supplement: Supplemental Material [file KEPI_A_2566505_SM7732.zip › SI-figures/Sup._Fig.4.JPG]

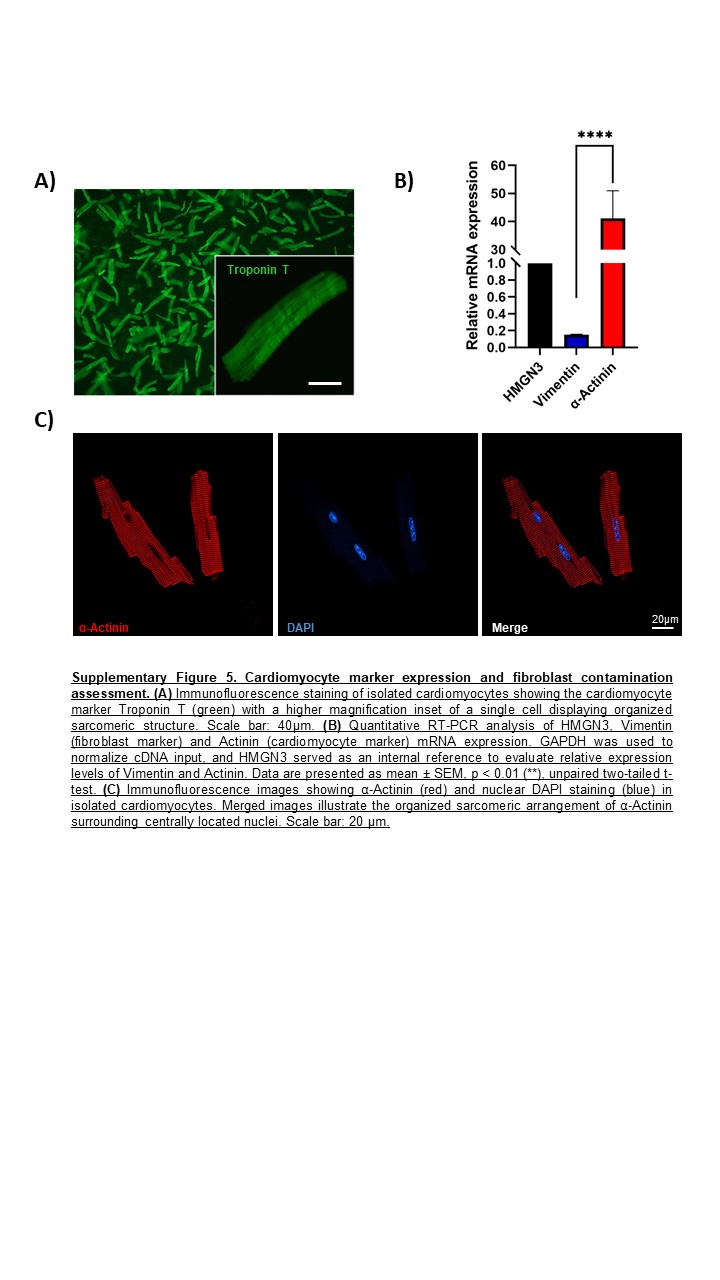

Supplement: Supplemental Material [file KEPI_A_2566505_SM7732.zip › SI-figures/Sup._Fig.5.JPG]

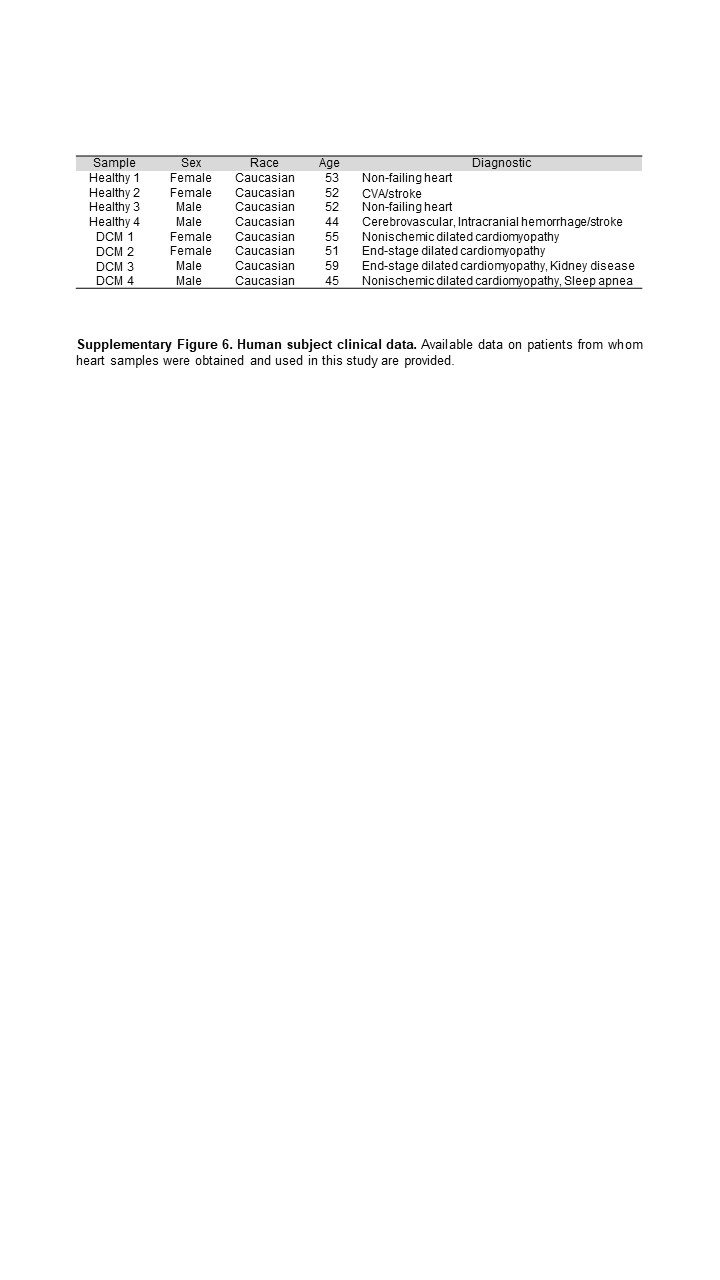

Supplement: Supplemental Material [file KEPI_A_2566505_SM7732.zip › SI-figures/Sup._Fig.6.JPG]

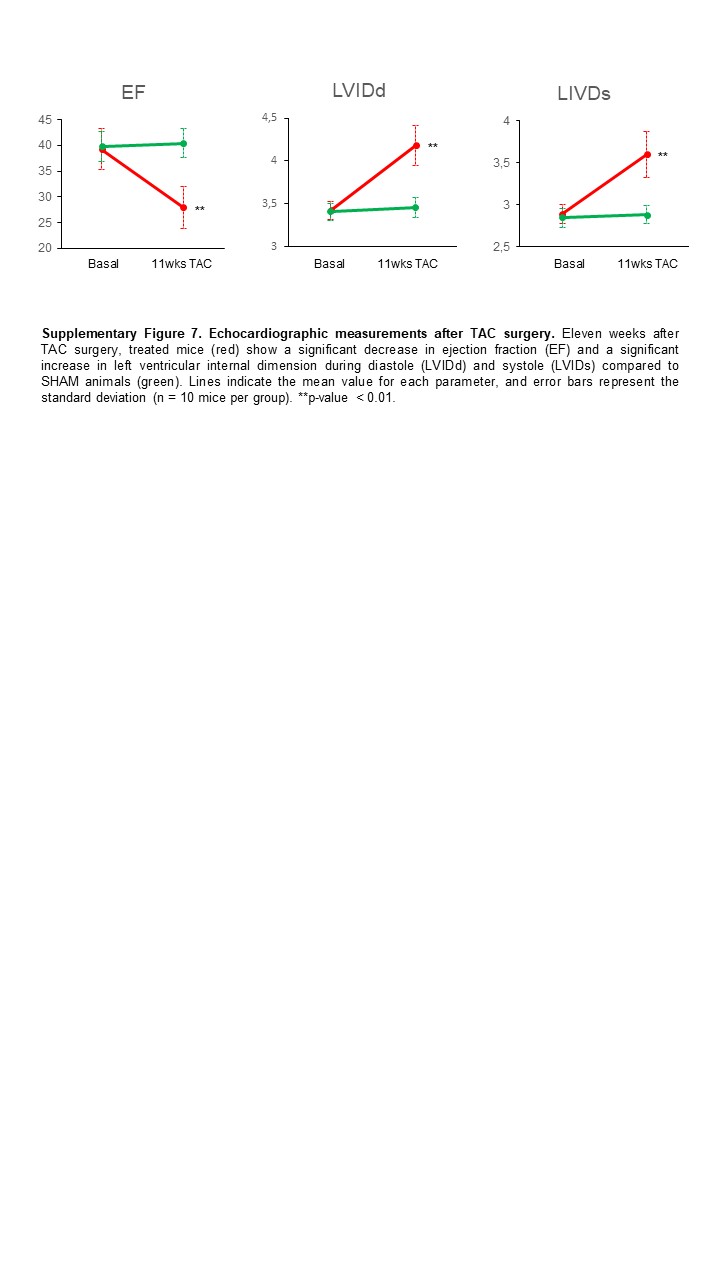

Supplement: Supplemental Material [file KEPI_A_2566505_SM7732.zip › SI-figures/Sup._Fig.7.JPG]

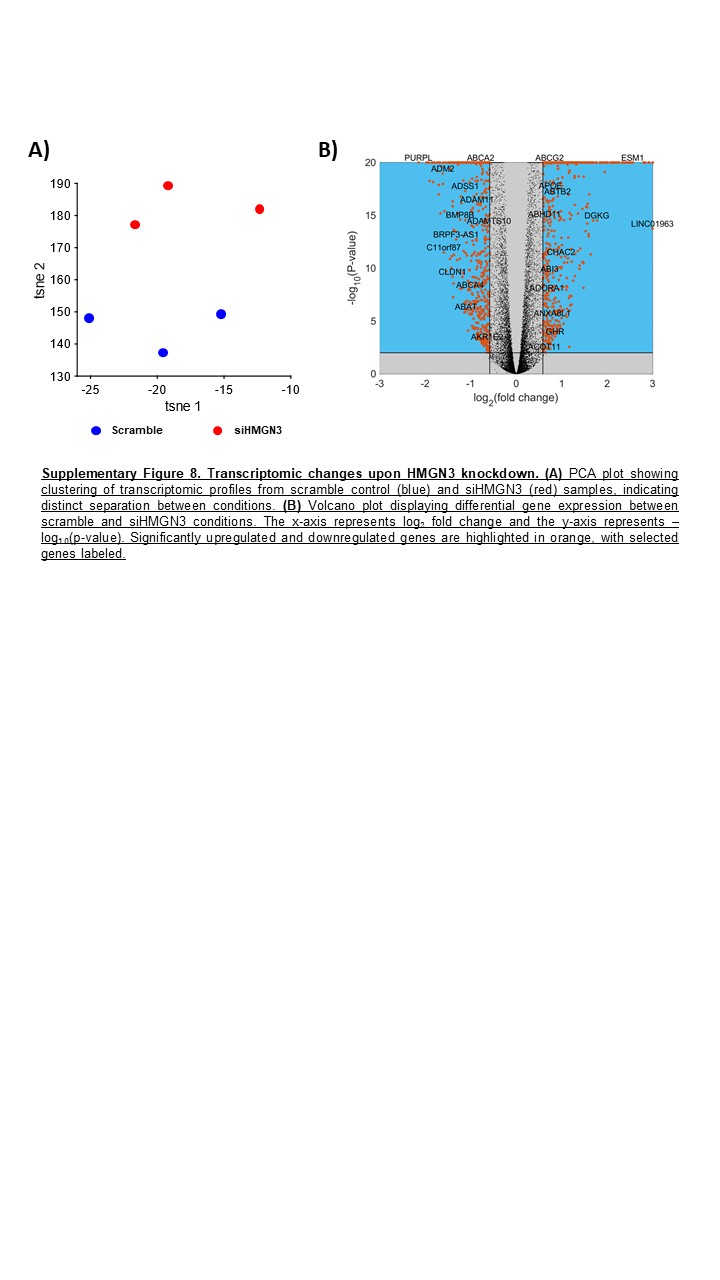

Supplement: Supplemental Material [file KEPI_A_2566505_SM7732.zip › SI-figures/Sup._Fig.8.JPG]

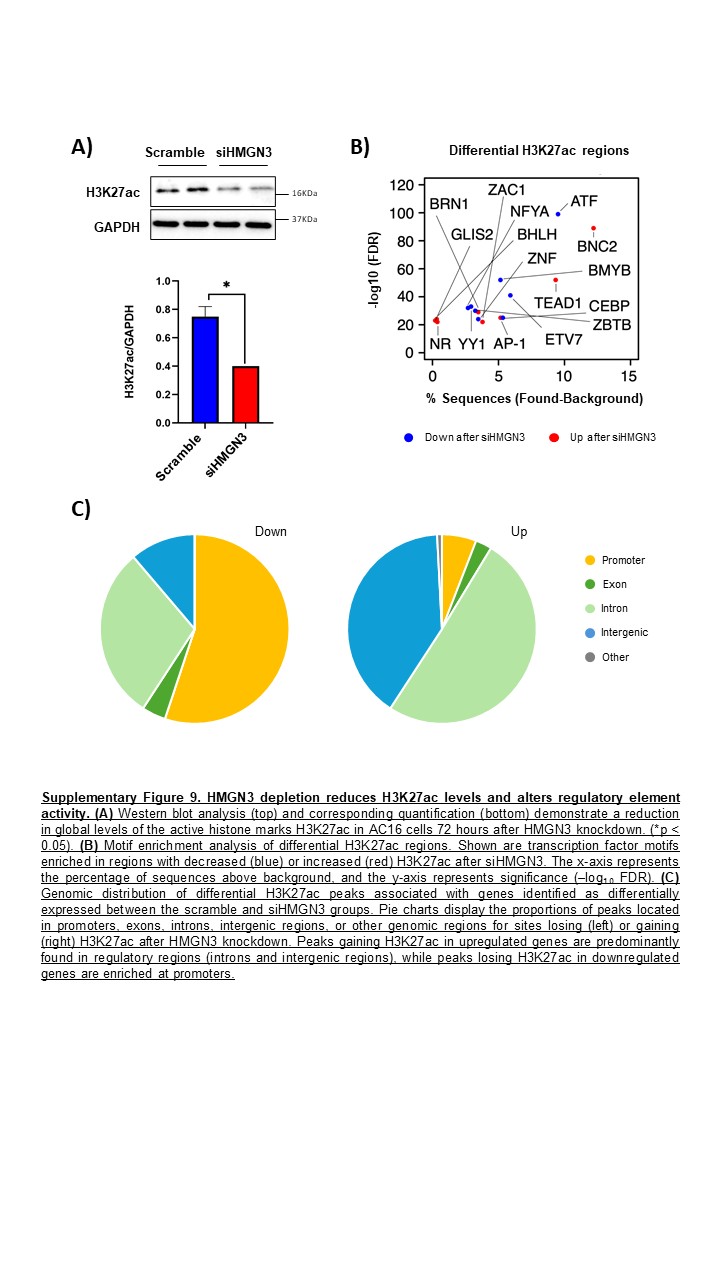

Supplement: Supplemental Material [file KEPI_A_2566505_SM7732.zip › SI-figures/Sup._Fig.9.JPG]
